# Phage therapy: What factors shape phage pharmacokinetics and bioavailability? Systematic and critical review

**DOI:** 10.1002/med.21572

**Published:** 2019-03-19

**Authors:** Krystyna Dąbrowska

**Affiliations:** ^1^ Bacteriophage Laboratory Institute of Immunology and Experimental Therapy, Polish Academy of Sciences Wrocław Poland; ^2^ Research and Development Center Regional Specialized Hospital Wrocław Poland

**Keywords:** bacteriophage, immune response, phage circulation, phage clearance, phage penetration, phage therapy, pharmacokinetics

## Abstract

Bacteriophages are not forgotten viruses anymore: scientists and practitioners seek to understand phage pharmacokinetics in animals and humans, investigating bacteriophages as therapeutics, nanocarriers or microbiome components. This review provides a comprehensive overview of factors that determine phage circulation, penetration, and clearance, and that in consequence determine phage applicability for medicine. It makes use of experimental data collected by the phage community so far (PubMed 1924‐2016, including non‐English reports), combining elements of critical and systematic review.

This study covers phage ability to enter a system by various routes of administration, how (and if) the phage may access various tissues and organs, and finally what mechanisms determine the courses of phage clearance. The systematic review method was applied to analyze (i) phage survival in the gut (gut transit) and (ii) phage ability to enter the mammalian system by many administration routes. Aspects that have not yet been covered by a sufficient number of reports for mathematical analysis, as well as mechanisms underlying trends, are discussed in the form of a critical review.

In spite of the extraordinary diversity of bacteriophages and possible phage applications, the analysis revealed that phage morphology, phage specificity, phage dose, presence of sensitive bacteria or the characteristics of treated individuals (age, taxonomy) may affect phage bioavailability in animals and humans. However, once phages successfully enter the body, they reach most organs, including the central nervous system. Bacteriophages are cleared mainly by the immune system: innate immunity removes phages even when no specific response to bacteriophages has yet developed.

## 1 INTRODUCTION

The burst of interest in bacteriophages as therapeutics, nanocarriers, or microbiome components makes bacteriophages no forgotten viruses anymore. In current times of emerging drug resistance in bacteria, bacteriophages are proposed as a new class of antibacterials, a serious alternative to antibiotics. Despite remaining outside the mainstream for decades, clinical applications and experimental studies of phages have been reported. Recently, a number of extensive reviews of phage safety, efficacy, and clinical history have been published.^1^, ^2^, ^3^, ^4^, ^5^, ^6^, ^7^, ^8^, ^9^, ^10^, ^11^, ^12^, ^13^, ^14^, ^15^ They support the applicability of phage therapy as an alternative to antibiotic antibacterials. However they also point out important uncertainties that could hinder development of modern phage therapy.

Fundamentals of phage pharmacokinetics in animals and humans are different from those of chemical drugs. They integrate the classical view of antibacterial pharmacokinetics with ecological aspects of phages as a self‐replicating element of microbial communities within the body, along with characteristic responses of the body to virions.^16^, ^17^, ^18^, ^19^, ^20^ Moreover, bacteriophage is a universal name applying to the apparently most diverse entities in the world.^21^, ^22^, ^23^, ^24^, ^25^ Factors that determine phage predisposition to successfully penetrate, circulate, or rather to be promptly cleared from a system are vague, due to the extraordinary diversity of bacteriophages and to the multiplicity of possible phage applications. Attempts to identify general rules face hurdles of a plethora of seemingly contradictory reports.

The purpose of this review is to summarize what can we learn from experimental data collected so far on phage circulation in animals and humans (vertebrates). The major topics cover: (i) phage ability to enter a system by various routes of administration (section 2), (ii) how (and if) the phage access various tissues and organs (section 3), and finally (iii) what factors determine the course of phage clearance (section 4). To foster unbiased conclusions, the review employs both critical and systematic analyses of experimental data collected so far by phage researchers (all available in PubMed, including those written in Russian, Georgian, German, and French). Specifically, the systematic review method is applied when the amount of available data is sufficient to allow for statistical analysis. This was found to be the case with (i) phage survival in the gut (herein: gut transit) and (ii) phage ability to enter the mammalian system (herein: phage penetration). A systematic analysis is presented in detail in the Supporting Information material. Aspects that have not yet been covered by a sufficient number of reports to allow for mathematical analysis, as well as mechanisms underlying trends, are discussed instead in the form of a critical review, based on a comprehensive summary of available experimental reports.

## 2 ENTERING THE SYSTEM

### 2.1 Gastrointestinal tract

Gastrointestinal tracts of animals and humans are natural environments for bacteriophages. The recent burst of metagenomic research has revealed an abundance of bacteriophage components of human and animal gut microbiomes.^26^, ^27^, ^28^ Yet even before that, traditional microbiological assays always revealed a multitude of gut phages. On the other hand, the oral route of administration is a very convenient way for delivery of various therapeutic agents. Since it is generally well accepted by patients, and it is considered relatively safe and easy, phages for therapeutic purposes are often applied this way, for targeting infections located in the gastrointestinal tract as well as other sites in the body.^1^, ^29^, ^30^, ^31^ In this section, two crucial aspects of phage oral delivery are discussed: (i) phage ability to survive when passing subsequent gut sections (gut transit), and (ii) phage effectiveness in entering systemic circulation and tissues after phage delivery to the gastrointestinal tract (Table S1 and S2).

Gut transit of active bacteriophages is typically assessed by the detection of active phages in feces after oral application. Among experiments analyzed in this review, 90 of 91 (99%) revealed successful gut transit for various bacteriophages (Table S1). Thus, bacteriophages commonly can travel through gastrointestinal tracts. Specifically, this was demonstrated in human safety studies^32^, ^33^, ^34^ and in animal models, including mice, rats, chicken, quails, calves, pigs, and sheep (Table S1). Although bacteriophages pass through the alimentary tract, their recovery from feces can be low. Sarker et al^33^ assessed the fecal recovery rate of T4‐like phages administered orally to 15 healthy volunteers in Bangladesh at less than 1%. Further analysis will be focused on factors that may affect gut transit of phages, increasing or decreasing the recovery of active phages.

**Figure 1:**
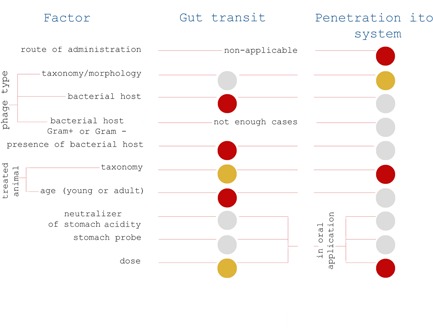
Factors affecting phage gut transit and phage penetration in animals and humans. Summary of systematic analysis; red—highly significant influence of a factor, yellow—significant influence of a factor, ānd gray—insignificant (Tables S1 and S2) [Color figure can be viewed at wileyonlinelibrary.com]

Reports on the dose effect on phage recovery show either a dose dependency of phage titer in fecal samples^32^, ^35^ or instead very similar degrees of phage shedding even when doses differed by as much as two orders of magnitude.^36^ Systematic analysis of all available reports (Table S1) revealed a significant, positive correlation between phage dose and recovery of the phage (*P* = 0.03654) (Figure 1).

**Figure 2:**
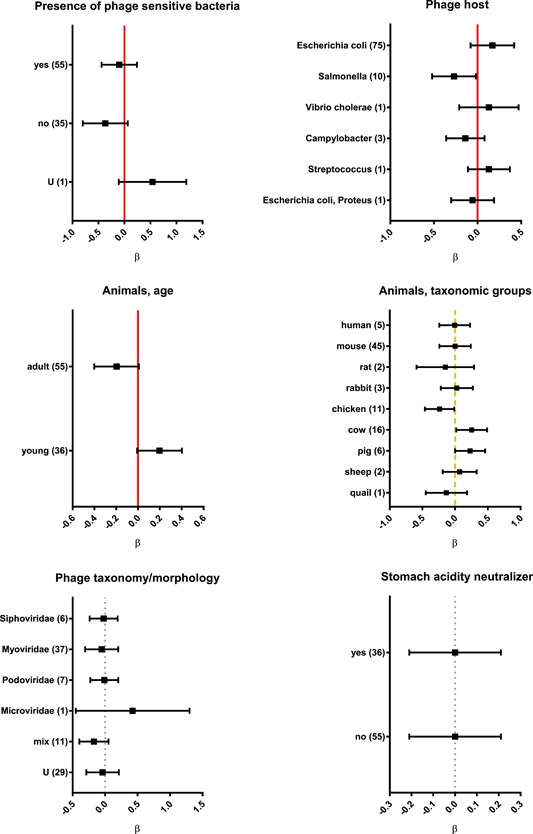
Factors affecting phage gut transit in animals and humans analyzed by categories, results of systematic analysis presented in forest plots. Vertical lines represent average effect within a category, horizontal bars represent confidence interval 95%; red (normal line)—highly significant influence of a factor, yellow (dashed line)—significant influence of a factor, gray (dotted line)— insignificant, U—unspecified, and ß—regression coefficient. Number of relevant reports available for analysis is given in parentheses. (Tables S1 and S2) [Color figure can be viewed at wileyonlinelibrary.com]

The gut transit and phage activity in the alimentary tract may depend on phage stability in all its sections, including the stomach and the intestine. Phages in particular are often considered to be unstable in the stomach and upper small intestine.^37^, ^38^ A factor that is commonly considered to be especially devastating to bacteriophages is extreme pH values, particularly the low pH in the stomach. Gastric acidity neutralizers have been commonly recommended to maintain phage activity in the alimentary tract,^38^, ^39^, ^40^, ^41^ and they have been routinely used in experimental therapy in humans.^30^, ^42^, ^43^, ^44^, ^45^ As demonstrated, the use of slightly alkaline mineral water, which did not ensure acidity blocking, resulted in effective gut passage of phages in animals^46^, ^47^ and in humans.^31^, ^32^, ^33^ Chibani‐Chennoufi et al^35^ found no correlation between stomach passage of a phage and concentration of bicarbonates in the phage vehicle. Also, successful gut passage without any acidity neutralization has been demonstrated in animals.^48^, ^49^, ^50^, ^51^, ^52^ Systematic analysis of available reports (Table S1) reveals a lack of significant improvement in fecal recovery resulting from the use of stomach acidity neutralizers in individuals treated orally with a bacteriophage (Figures 1 and 2). This is in contrast to common expectation and thus will be discussed further below.

Bacteriophages are generally considered to be acid‐sensitive, but they differ strongly in their response to acidity. There are phages that are highly sensitive even to moderate pHs, but others which are quite resistant to even very low pHs. This topic was recently reviewed by Jonczyk et al.^53^ Methodological differences between experiments (such as time of exposure, temperature, and ions) substantially impede comparisons, but most bacteriophages are relatively stable in neutral or near‐neutral conditions. As to a “phage acceptable” pH range, more sensitive phages survived only at pH 6.0 to 8.0 (eg PM2 phage) while others survived at pH 3.0 to 11.0 (eg, lambda phage).^53^ Even when a phage demonstrates decreased activity at low pH, this can be a reversible effect related to aggregation or other physical effects.^53^ Notably, sensitivity of phages to extreme pHs is typically investigated in vitro, thus excluding the potential impact of ionic composition and variety of organic (potentially protective) macromolecules present in the stomach. Therapeutic phage can be protected by encapsulation for example in polymeric microparticles, liposomes, or others.^54^ In vitro exposition of encapsulated phages to low pH clearly demonstrated that they were more resistant to acidic environment than free bacteriophages, some data indicate that encapsulation improve phage antibacterial activity in vivo.^54^, ^55^, ^56^, ^57^ Thus, at least in phage strains that are highly sensitive to acidic environment, encapsulation may protect them during gut transit.

The pH in the human stomach lumen is the lowest among all species that have been analyzed in this review, and can be as low as 1.5 (Table S3). The normal range is from 1.5 to 3.5 and in a fed person it can reach 5.0,^58^, ^59^ thus in the same person a bacteriophage can either be inactivated or not, depending on the particular conditions. The pH of the intestinal lumen in humans, in turn, can be acceptable for virtually all phage strains: it is about pH 6 in the duodenum, then gradually increases to about pH 7.4 in the terminal ileum. In the cecum the pH drops to about 5.7 and becomes almost neutral, reaching a pH of 6.7 in the rectum.^60^ In mice, the most common species used for in vivo phage studies, stomach acidity is weaker than in humans: pH values do not drop lower than approximately 3.5. In other sections of the gastrointestinal tract they range from about 4.4 to 5.2; similar values can be observed in rats.^61^ These differences between humans and model animals should be taken into account, since bacteriophages intended for application in humans are often evaluated first in rodents. The analysis herein (Table S1; Figure 2) reveals differences between taxonomic groups of animals (*P* = 0.0488) and between young and adult individuals. In young individuals, phage recovery was significantly higher than in adults (*P* = 0.0257) (Figures 1 and 2). These differences are probably related to variations in the physiology of the gastrointestinal tract, possibly including the pH range, but also those related to digestive compounds and bacterial microflora.

Gut transit and phage recovery in feces can be facilitated by phage propagation on sensitive bacteria, if those bacteria are present in the gut. This may result either from bacterial infections (including experimental ones) or from the presence of natural symbionts within gut flora. Systematic analysis consistently indicates that introduction of a bacterial host to an investigated system can increase phage recovery from feces (Figures 1 and 2; Table S1). Phage T4 propagation in the alimentary tract was, in fact, demonstrated in mice that had simultaneously received the phage along with a sensitive *Escherichia coli* (*E. coli*) strain. Phage titer was lowest in the stomach, but it increased approximately 1000‐fold in the duodenum, jejunum, and proximal ileum, and a further 10 to 100‐fold in the distal ileum and in the large intestine.^47^ Wagenaar et al^62^ demonstrated in chickens that phage maintenance in the gastrointestinal tract depended on the presence of the bacterial host. In general, phage propagation on infecting bacteria was observed in cases where infections were successfully controlled by phages for example with *E. coli* infections of pigs and calves.^40^, ^63^ Poor phage propagation correlated with ineffective control of infection in animals and in humans.^31^, ^41^, ^64^, ^65^, ^66^

In the case of nonpathogenic symbionts present in the gut, some observations made it questionable whether phages could effectively propagate on these bacteria, for example T4 phage did not propagate in healthy animals with sensitive symbionts.^46^, ^50^ Weiss et al^46^ showed that T4 did not but T7 massively propagated under the same conditions, while T4 and T7 differed in their abilities to grow in stationary phase bacteria. Therefore, physiological state of symbiotic bacteria may limit propagation of those bacteriophages that are not able to propagate effectively in stationary phase cells for example T4.^46^, ^67^ However, this limitation does not rule out the impact that host range of the applied phage has on its gut passage in general, as identified by the systematic analysis herein (Figure 1). Detailed analysis showed that coliphages were characterized by the highest recovery rates (Figure 2). Coliphages are able to propagate on *E. coli* which is a common symbiont that may be present in the gut even without deliberate introduction. This suggests that for bacteriophages the potential to propagate during their gut passage can have a major effect on phage recovery, and that it can outweigh phage loss resulting, for instance, from stomach acidity. Notably, phage replication on gut bacteria increases phage recovery, but it is not obligatory for successful transit of the phage. This was shown in germ‐free mice, where a T4‐like phage was administered in drinking water and it was recovered from feces, in spite of the absence of intestinal bacteria.^35^

Phage ability to replicate in the gut may pose a selective pressure on gut bacteria favoring phage‐resistant mutants. Probably, intensive phage replication correlates with a short time of selection for resistant bacteria.^37^ In models with a lesser impact of phage replication, selection for resistant bacteria can take a long time, even more than 90 days of exposure to phage, while having no important effect on the phage gut transit itself.^50^ Possibly the “arms race” between phages and bacteria in laboratory liquid monocultures is much more rapid than in the complex environment of the mammalian gut. Maura and Debarbieux^48^ did not detect phage‐resistant mutants during 30‐day exposures of animals to the phages, even though they observed phage propagation in the alimentary tract. Therefore, they proposed that the phage‐resistant mutants might have had no selective advantage in the gut environment.

Contrasting to phage host specificity, phage morphology, and related taxonomy seem to be of no significance for phage passage through the gut. No consistent differences between phages belonging to different groups *(Myo‐*/*Podo‐*/*Siphoviridae)* were observed (Figure 1 and 2), thus demonstrating that general physical properties of phage particles had no important effect on gut transit.

Other factors that may affect phages inside the gastrointestinal tract—digestive enzymes and bile—have only been investigated in vitro/ex vivo. These studies have shown that bacteriophages were relatively resistant to digestive enzymes, but with differences between phage strains. Four tested enterobacteria phages (T4, T2, UZ1, lambda) and one *Vibrio cholerae* phage were resistant to trypsin or to whole pancreatic juice,^39^, ^68^, ^69^ while coliphage P1 was sensitive to this enzyme.^69^ Interestingly, T4 phage was quickly inactivated by trypsin when heated to 60°C. This effect was correlated with structural changes in the phage proteins responsible for phage adsorption to bacteria. This may suggest that at least some gut bacteria‐associated bacteriophages have evolved a level of resistance to digestive enzymes, probably by losing or hiding molecular elements that can be recognized by these enzymes. Heating enhances exposure of these elements and makes them more accessible to proteolytic enzymes.^70^ Notably, other proteases such as proteinase K or papain can easily inactivate T4 phage.^71^, ^72^

Bacteriophages do not seem to be easily inactivated by bile. As demonstrated in a few *Enterobacteriaceae*‐infecting phages, incubation with bile acids, bile salts, or porcine bile extract had no or only a moderate effect on the phages.^51^, ^73^, ^74^ All these suggest rather that phages tend to be bile‐resistant. Maura et al^75^ found that three different bacteriophages related to phages T4, T7, and T1 were not inactivated by intestinal homogenates ex vivo (that are rich in bile),^76^ thus demonstrating that the inhibitory effect not only of bile but also other agents secreted to the intestine is questionable. This might further suggest that phages linked to gut symbionts have developed resistance to bile, but the KPP10 phage specific to *Pseudomonas* was also found to be resistant to cholic acid (a primary bile acid).^77^

Another factor that can neutralize phages in the gut is the host immune response. Smith et al^63^ noted in calves that oral delivery of phages together with its specific antibodies resulted in a substantial reduction of active phages in the intestine. Majewska et al^50^ demonstrated in mice that the main factor limiting gut passage of T4 phage was induction of secretory IgA, where a significant increase of specific IgA antibodies in feces correlated with the complete loss of active phage. Although specific IgG in blood was also observed, it had no neutralizing effect on gut transit of the phage. Phage immunogenicity, i.e. its ability to induce antibody production, was concluded in that report to be low. This was due to the high doses and very long time of exposure that were necessary to induce antibodies.^50^

**Figure 3:**
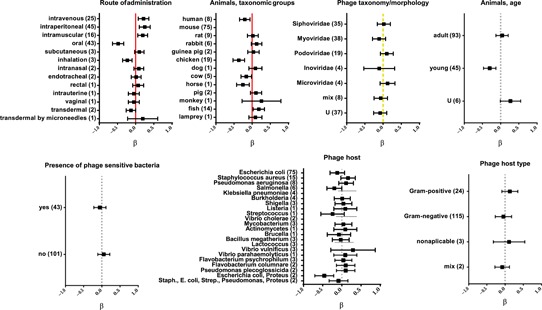
Factors affecting phage penetration in animals and humans analyzed by categories, results of systematic analysis presented in forest plots. Vertical lines represent average effect within a category, horizontal bars represent confidence interval 95%; red (normal line)—highly significant influence of a factor, yellow (dashed line) –significant influence of a factor, gray (dotted line)—insignificant, U—unspecified, and ß—regression coefficient. Number of relevant reports available for analysis is given in parentheses (Tables S1 and S2) [Color figure can be viewed at wileyonlinelibrary.com]

Phage ability to pass from the gastrointestinal tract to systemic circulation (phage penetration) is the major issue regarding the oral route of phage administration. Available reports either show its efficacy or raise a question mark over oral application of phage. Thus, in this review, various routes of application were subjected to systematic analysis and compared. One hundred forty‐four relevant experiments (Table S2) were classified according to zero‐one characteristics, i.e. indicating that active phage was (1) or was not (0) detected in any investigated organs or tissues at any time (for details see the Supporting Information material: methods). The analysis demonstrated that delivery route had a major effect on phage ability to penetrate into the system (Figures 1 and 3). Among all routes of administration, oral delivery was the worst. It was the least effective among other, namely intraperitoneal (IP), intravenous (IV), or intramuscular (IM) routes (*P* < 0.001). Notably, all observations of ineffective phage delivery among 144 analyzed experiments were those when the phage was delivered per os (Figure 3, Table S2). Only 20% (9 of 43) of oral applications were fully efficient in all investigated individuals, while 33% (14 of 43) resulted in no penetration in any individuals of a studied group (Table S2). Those ineffective applications included, for example, a safety trial of healthy human volunteers with T4 phage applied orally. No active bacteriophage was detected in blood, although gut transit of the active phage was confirmed.^32^

**Figure 4:**
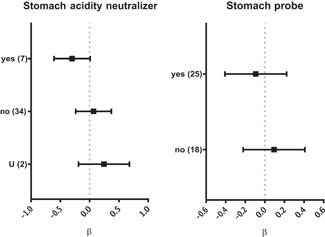
Selected factors specific for phage penetration after oral administration, results of systematic analysis presented in forest plots. Vertical lines represent average effect within a category, horizontal bars represent confidence interval 95%. No statistical significance was observed (gray, dotted line). U—unspecified and ß—regression coefficient. Number of relevant reports available for analysis is given in parentheses (Table S2)

Since the oral route of administration has been revealed as a difficult one, factors that may specifically influence its efficacy—stomach acidity neutralizers, use of a stomach probe, and phage dose—were analyzed. Similarly to what was observed in the case of gut transit, use of stomach acidity neutralizers did not significantly increase the penetration of orally administered phages ( Figures 1 and 4). Stomach probes or even surgical infusions are used instead of free uptake to ensure dosage control and to prevent spread of phages to animals and equipment. Stomach probes, surgical infusions, or other types of force feeding, however, pose a risk of mock delivery of phages to blood by microinjuries or wounds. According to the systematic analysis herein, experiments where they were used did not result in any significant increase in systemic delivery of phage (Figures 1 and 4). Hoffmann^78^ pointed out that a stomach probe may even decrease the efficiency of systemic delivery; he detected more phages in blood of mice fed with phages (68%) than in those treated with a deeper stomach probe (23%) or with an esophageal probe (20%). Moreover, phages were still able to reach the blood in 90% of animals in which stomachs was blocked and phage absorption in the intestine was not possible. This may suggest that a portion of orally administered phages contaminate the respiratory tract and penetrate from there.^78^

**Figure 5:**
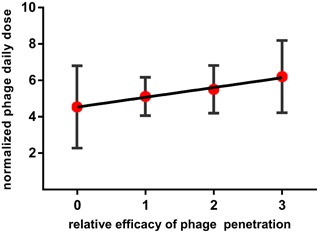
Correlation between phage dose in oral administration and the efficiency of phage passage to blood. ‐0, phage was not detected in any individual, 1, phage detected in not more than 50% of individuals, 2, phage detected in more than 50% but less than 100% of individuals, 3 to 100% of individuals were positive for a phage (calculated from all 43′oral‘ experiments presented in Table S2) [Color figure can be viewed at wileyonlinelibrary.com]

The factor that, according to the systematic analysis presented herein, significantly contributed to phage penetration after oral administration was the dose, as calculated from dose normalized per gram of body weight (*P* = 0.0185) (Figure 5). Dose dependency was directly demonstrated in rodents and in chickens, where the minimum effective doses seemed to be around 10^7^‐10^9^ plaque‐forming units (PFU) per animal.^50^, ^79^, ^80^ The relatively low oral phage dose (4.5 × 10^5^ or 4.5 × 10^7^ PFU daily) that was used due to ethical approval issues in the human safety trial did not allow active T4 phages to be detected in patient blood.^32^ Monsur et al,^81^ who used phage treatment against cholera in humans, showed that, due to high phage doses (10^13^‐10^14^ PFU) and phage propagation, phage concentration above 10^11^ PFU per gram of feces was achieved. In the same patients, they observed only approximately 10^2^ PFU per mL of patient blood. Thus, the overall rate of phage absorption in the gastrointestinal tract seems to be very low.

One should note that in most experiments there are detection limits for active phages, which typically vary between 50 and 200 PFU/mL or g. In human safety trials with T4 phage, it was 10 PFU/mL.^32^ These limits are derived from ethical issues and/or technical limitations of blood and tissue volumes that can be collected. Low phage penetration and its dilution in blood volume may result in undetectable phage titers, even if rare phage particles are able to penetrate to organs. These phages, however, if they reach a site of bacterial infection, can propagate on susceptible bacteria, increasing the phage amount in situ. Orally delivered phages can be effective in phage therapy in many body sites distant to the alimentary tract.^29^, ^30^ For example, in 58.3% of patients with orthopedic infections treated with bacteriophages (Phage Therapy Unit, Hirszfeld Institute of Immunology in Wrocław, Poland) showed a good response that was related to oral application, whereas with topical application the response rate was only 20%.^30^

Seeking to understand the mechanism of phage absorption in the gastrointestinal tracts, one may consider two aspects of the movement of molecules across the gut wall: (i) intestinal permeability and (ii) intestinal transport. Both aspects can be discriminated depending on physical parameters of penetrating molecules. Intestinal permeability allows non‐mediated passive diffusion and it applies to ions and inert small molecules, and thus not to phages. Intestinal transport comprises paracellular diffusion through tight junctions between epithelial cells and transport by transcytosis (exocytosis/endocytosis).^82^ The dimension of the paracellular space in the natural state ranges from 0.3 to 1.0 nm; even when fully opened, the tight junction space is less than 20 nm,^83^ seemingly too narrow for most phages. On the other hand, eukaryotic viruses can probably sneak into tight junctions. Phage entry by this pathway thus is not definitely excluded and still needs to be investigated. Low internalization of bacteriophages by enterocytes and other epithelial cells was first demonstrated on M13 phages in vivo (empty vectors used as a control in phage display) by Costantini et al^84^ and in vitro by Ivanenkov et al^85^ Since the in vitro uptake was blocked by chloroquine, which inhibits clathrin‐dependent endocytosis, this pathway was proposed as the mechanism.^85^ However, it is unclear how the M13 phage particle (7 × 900 nm) meets size restrictions for particles that can be uptaken by clathrin‐dependent endocytosis (less than 200 nm in diameter).^86^ Epithelial transcytosis of phages was recently observed in vitro by Barr et al.^87^ Transcytosis has been proposed as the major way of phage absorption in the gastrointestinal tract.^87^ The general ability of microorganisms to translocate from the gut to systemic circulation has been well documented, and it is determined not only by microbial size, but also by other factors for example presented molecular patterns. These have not been identified yet in phages. This may suggest macropinocytosis as the major endocytic pathway, since macropinocytosis does not require specific receptors with pattern recognition on engulfed objects.

After translocation, phages probably reach lymphatic pathways.^79^, ^88^, ^89^ This is in accordance with the biodistribution of large nanocapsules (drug carriers) that can pass from the gastrointestinal tract to blood and organs via the lymphatic system.^83^, ^90^ Phages may also travel only to intestinal lymph nodes and very few if any reach the blood, as observed in mice by Smith et al^63^ Lymph nodes act as filters with a 70‐kilodalton cutoff for molecules entering the parenchyma and high endothelial venules,^91^ whereas most phages are measured in megadaltons (eg T4 phage head: 194 MDa). Lymph nodes therefore likely constitute filters which can prevent orally administered phages from traveling further.

### 2.2 Respiratory tract

Bacteriophage delivery to systemic circulation via the respiratory tract reported so far includes inhalations, intranasal applications, and endotracheal applications (Table S2). None of these was significantly better or worse in comparison to other application routes, but they seem to be generally less effective than injections, with the least efficacy observed for inhalation (Figure 3). Bogovazova et al^92^ reported good intranasal phage delivery in mice (although no specific data were presented in that report). Mice and chicken models revealed poor phage delivery by inhalation in comparison to IP and IM injections, either to the lungs only or to systemic circulation. The authors found it questionable whether phages delivered to the airway were able to effectively penetrate the respiratory epithelium and to fight bacteria that have translocated from the lumen into the lung interstitium.^93^, ^94^

An unsolved issue is the possible contamination of phages delivered to the respiratory tract also to the gastrointestinal tract. The anatomy of animals and humans makes it likely that phages cross‐contaminate the airways and the alimentary tract. Endotracheally administered phage has been found in the esophagus,^95^ so its further detection in internal organs might result from penetration through either the lungs or the intestine. Systematic analysis of available reports shows that average efficiency of oral delivery of phage was less efficient than delivery via the respiratory tract (Figure 3). Also Hoffmann^78^ found tracheal application of T3 phage to be very effective as it allowed for approximately 1 order of magnitude higher titer of the phage to the blood than that after oral delivery. Therefore, the possible contribution of alimentary tract contaminations, at least in this case, seems to have low importance in phage penetration via the respiratory tract.

Even though systemic phage dissemination from the airways is probably not very efficient, phages were demonstrated to reach the lungs by inhalation^95^ and in most cases they were capable of controlling bacterial infections in the lungs. Inhalation with phages had a life‐saving effect in hemorrhagic pneumonia caused by *Pseudomonas aeruginosa* in a mink model,^96^, ^97^ *Acinetobacter baumannii*‐ or *Klebsiella pneumoniae*‐induced pneumonia in mice,^98^, ^99^ and fatal *E. coli* respiratory infection in chickens.^100^ Experimental pneumonia in mice was reduced by phage therapy in a time‐ and dose‐dependent manner.^101^ Very effective clearance of respiratory tract infecting bacteria was visualized by the group of Debarbieux.^102^, ^103^

### 2.3 Skin

Bacteriophages bearing large proteinaceous capsids are generally chemically distinct from drugs, which instead tend to be small lipophilic molecules. For this reason, phages do not satisfy the criteria for efficient transdermal absorption, in contrast to many “classical” pharmaceuticals.^104^ The scarcity of data, however, does not allow for a systematic analysis of transdermal delivery of bacteriophages. Nevertheless, as an example, early studies of a *Bacillus megaterium* phage transit through intact skin in mice showed low and irregular (not in all individuals) phage penetration to the blood.^105^ Bennet and Foster^106^ reported that after 15 minutes immersion of murine tails in streptophage lysates, phages were found in the blood of about 40% of mice. Tails were assessed macroscopically to avoid blood contamination with the phage, but the authors did not state that mice were prevented from licking their tails. Recent studies of random phage display libraries showed poor skin penetration of filamentous phages in rats (application of 10^12^ PFU per rat resulted in only 6.8 PFU per mL of blood after 1 hour).^107^ These reports suggest that phage transit through intact skin may be possible but not efficient. It may also depend on individual properties of phage particles. These can be engineered for better penetration properties for example by presentation of targeting peptides on the phage surface.^107^

In spite of inefficient phage transport through intact skin, phage penetration could possibly be enhanced in wounds. Studies of phage therapy in wounds, however, have not included assessment of systemic dissemination of phages. The investigators focused on bacterial load and phage propagation in the wound, monitoring of possible adverse effects, and on clinical improvement, which were found promising.^108^, ^109^, ^110^

Transdermal delivery of bacteriophages has been markedly increased by the use of microneedles, as demonstrated by Ryan et al^104^ In an in vitro porcine skin model and in vivo in rats, T4 bacteriophage effectively penetrated through the skin by the use of a microneedle device. In vivo, microneedle‐mediated transdermal application of 4 × 10^9^ PFU per rat resulted in 4 × 10^3^ PFU per mL of blood within 30 minutes after application. The authors concluded that the stratum corneum is the main barrier to phage delivery and once this barrier is breached the viable epidermis is not an important barrier to penetration.^104^

### 2.4 Injections

Injections are commonly used to deliver bacteriophages systemically, mostly in animal models. Phages have been injected intraperitoneally (IP),^111^, ^112^, ^113^, ^114^, ^115^, ^116^ intramuscularly (IM),^117^, ^118^, ^119^, ^120^, ^121^, ^122^ subcutaneously (SC)^113^, ^123^, ^124^ or directly into the blood intravenously (IV),^125^, ^126^, ^127^, ^128^, ^129^, ^130^ including IV administration to humans.^131^, ^132^ All types of injections have been demonstrated as efficient means of phage delivery to virtually all organs and tissues. Systematic analysis herein revealed that IP, IM, and IV delivery routes were significantly better than oral delivery (*P* < 0.001) (Table S2).

Injections are not only efficient, but also very fast in terms of phage delivery. Typically, active phages can be observed in the circulation within the first hour (even less than 5 minutes).^92^, ^133^, ^134^, ^135^, ^136^, ^137^, ^138^ IP injection was demonstrated as markedly (3 hours) faster at delivering phage than oral application. Also, the maximum phage titer in the blood was reached faster (6 hours) after IP delivery than after oral one.^139^ IP injection allowed for faster arrival of phages in the blood compared to IM or SC injections; it also resulted in higher phage titers in the blood as well as more effective protection of mice from lethal septicemia.^124^

Injections of bacteriophages are highly applicable in therapies against bacterial infections, either systemic or localized ones.^123^, ^140^ Systematic analysis herein did not reveal that presence of sensitive bacteria significantly facilitates phage entrance into animal or human bodies (Figure 1). Notably, this analysis was of the yes/no type, not considering phage titers that could be reached in tissues due to, in most cases, noncomparable data (for details see the supplementary material: methods). Nevertheless, there are reports documenting much higher phage concentrations in infected animals than in healthy ones, by up to 6 orders of magnitude (IP and IM injections).^114^, ^115^ Matsuzaki et al^115^ found that shortly after IP injection of phage (2 hours), the phage titer in blood was lower in infected animals, but it increased later (6 hours). This effect was attributed to the initial “binding” of free phages by bacterial cells, thus preventing their fast spread throughout the system. Later the phage completed its amplification in bacteria and its systemic titer increased.

## 3 TRAVELING INSIDE THE SYSTEM

### 3.1 Phages in the blood

Bartell et al^135^ demonstrated the dose dependency of phage concentration in murine plasma after systemic delivery of the phage, both in the presence and in the absence of sensitive bacteria. As to the basis of this dependency, bacteriophages are expected to be diluted in the body, possibly in the whole body volume (relative to body weight), or at least in blood volume when delivered IV. It was noted as far back as 1957^138^ that phage concentration in the blood of rabbits immediately after injection (2.5 minutes) was 100 folds lower than the hypothetical titer calculated from the phage dose and its dilution in blood volume. Other studies gave different results.^126^, ^127^, ^129^, ^141^, ^142^ The combined analysis (Table S4) shows that immediately after phage IV injection (1‐5 minutes) the median phage titer in blood was 0.02 of the hypothetical value calculated from the phage dose and its dilution in the blood volume. Half an hour after injection it was approximately 0.003 (Table S4). Even assuming that phage was able immediately dilute in the whole body (not only in blood that in mammals is approx. a total of 5% of body volume), the respective values were only 0.4 (1‐5 minutes) and 0.068 (0.5 hours). This is markedly less than expected from the hypothetical dilution of phage in body volume and it suggests a very rapid mechanism of capturing or neutralizing the phages. As demonstrated in rodents, titers of IV injected phages drop most rapidly within the first 30 minutes, approximately 1 to 2 orders of magnitude, though later this decrease is usually much slower.^127^, ^129^, ^138^, ^141^, ^143^

Thus, when reaching systemic circulation, phages not only are diluted, but also can be immediately captured or filtered, probably by the mononuclear phagocyte system (MPS; older term: reticuloendothelial system, RES), as proposed by the early researchers and confirmed later.^19^, ^127^, ^144^, ^145^, ^146^ Other blood cells that have been proposed as able to bind bacteriophages are erythrocytes^147^; nevertheless, their contribution to possible capturing and circulating of phage particles is not well documented and remains unclear. Earlier studies along with our experiments with T4‐like phages applied systemically have not revealed phage binding to animal erythrocytes^148^ (Dąbrowska et al, unpublished data).

### 3.2 Phages in organs

Liver and spleen are the most commonly reported organs that accumulate bacteriophages delivered systemically (Table S2). As important elements of the MPS, they filter many foreign objects traveling in circulation, including phages. They were identified as the major sites of phage accumulation in early studies. Phage titers are usually the highest there, even higher than in the blood.^120^, ^125^, ^127^, ^149^ After systemic delivery, phages can reach spleen and liver within minutes^150^, ^151^, ^152^ and attain relatively high titers within 1 to 3 hours.^120^, ^122^, ^149^ Typically, spleen is the organ where active phages can be detected for the longest time, even for many days after administration.^121^, ^147^, ^153^ Different phages may achieve different concentrations in the same experimental model,^80^ which suggests strain‐specific capabilities of phage accumulation by MPS.

Other organs settled with cells constituting the MPS, such as lymph nodes, are less often reported as able to filter bacteriophages (Table S2). Some reports have demonstrated a lower titer (approximately 2 orders of magnitude lower) of phages in lymph nodes than those in spleen and liver.^19^ On the other hand, oral administration of bacteriophages in Smith et al^37^ resulted in phage accumulation in intestinal lymph nodes, while a very small titer was detected in blood samples and in the spleen. This suggests that intestinal lymph nodes may contribute to the differences between administration routes in their efficiency in systematic delivery of phages.

Other organs where phages have been detected after systemic delivery are skeletal muscles, heart, thymus, bone marrow, kidneys and bladder (Table S2). One report also demonstrated rapid phage penetration to the salivary glands and saliva in mice (the animals were protected from licking the site of administration).^78^ All these organs do not appear to accumulate phages, but clearly they can be reached by phages, and thus infections localized in those sites can potentially be controlled by phage therapy. For this purpose, further studies including penetration of these organs by particular phage strains will be important. Hypothetically, phage arrival at those sites may depend on individual molecular features of phages and on the dose and duration of phage circulation in the whole system.

Brain penetration by bacteriophages provokes probably the most exciting discussion, due to the existence of the physiological blood‐brain barrier. The brain is considered to be accessible for nutrients and oxygen but potentially challenging for delivery of drugs.^154^ Whatever problems could be envisaged concerning physical properties of phage particles, many researchers who have delivered phage to blood (by various administration routes) have also detected active phages in animals brains; this was demonstrated for a few morphologically different groups of bacteriophages: *Myoviridae*, *Siphoviridae*, and *Podoviridae*.^95^, ^113^, ^115^, ^121^, ^149^, ^155^, ^156^ Capability of systematically administered phages to control intracerebral infections was shown by Dubos et al^156^ They observed increased phage titers in the brains of animals with intracerebral infection with phage‐sensitive bacteria, which suggested effective phage multiplication in the brain.

Major conditions that influence phage penetration to the brain seem to be dose, duration of phages within circulation, and delivery route. In the study of Schultz and Frohlich^142^ in dogs, phage titer in cerebrospinal fluid correlated with blood concentration of the phage, which in turn correlated with phage dose injected IV (*P* < 0.03, the Mann‐Whitney *U* test, calculated by the author of this review from raw data given by Schultz and Frohlich). Recent studies of blood/tissue ratios in rats suggested that shortly after IP administration (2 and 6 hours) most phages detected presumably originated from the blood, but later (24 hours) phages had effectively crossed blood‐brain barrier.^113^ In addition to systemic delivery routes, intranasal administration allowed for filamentous phage delivery to the brain in mice. The phage particles were visualized by immunofluorescence in the olfactory bulb and in other regions. Phages were not present in the cortex, but the authors highlighted dose dependency of phage penetration. The filamentous shape of the phages seemed to be crucial for their ability to access the brain, since the chemically induced spheroid forms of the same phage did not penetrate by intranasal administration.^157^

Increasing phage delivery to the brain by a convection‐enhanced method was tested in primates. This method comprises surgical placement of an infusion pedestal and then the infusion of a studied preparation. Filamentous phage M13 was successfully distributed this way and visualized by immunostaining in monkey brains (*Macaca mulatta*). The phage was detected in grey matter as well as white matter, but in the thalamic grey matter the phage was contained in the discrete region of perfusion. In white matter, most of the phage was detected in the region of perfusion, but it also emanated from the site of infusion to the adjacent fiber tracts and through the corpus callosum. The authors concluded that axonal transport allowed for phage distribution in white matter.^158^

Airways can be treated with phages directly by inhalation, intranasal application, or by delivery in situ for example endotracheal. These ways of delivery have resulted either in demonstrated phage presence in airways or in controlled experimental infections.^95^, ^100^, ^103^, ^118^, ^133^, ^159^ Carmody et al^94^ showed in mice that bacteriophage BcepIL02 inhaled intranasally colocalized with alveolar macrophages. This suggests that a large portion of phage delivered intranasally was inactivated, but the phage was still effective against infection (MOI = 100 applied).

Phages delivered systemically may also reach the lungs in healthy animals.^155^ Singla et al,^93^ who studied *Klebsiella pneumoniae*‐induced lobar pneumonia in mice, observed IP administered bacteriophages primarily in vascular and perivascular areas and along alveolar septa. Phage ability to penetrate to the alveolar cavity was, however, questioned by Takemura‐Uchiyama et al,^114^ who reported that bronchoalveolar lavage fluid (BALF) contained a lot of phages only in infected animals and no phage was present in noninfected ones. They concluded that diffusion from the bloodstream into the alveoli could be facilitated by the pores created by bacterial invasion. Semler et al^112^ successfully controlled bacterial pulmonary infection by inhalation with phages but not by IP administration. Also, Carmody et al^94^ found significantly greater titers of phages in lungs after treatment via inhalation than delivery via the IP route, which suggests that phage penetration from blood to pulmonary cavities is poor. Unexpectedly, they observed that IP injected phages were still better in controlling pulmonary infection. To explain this, they proposed that circulating phages had better access to bacteria in the lung compared to topical phages. Other authors pointed out that the systemic dose of phage needed to be relatively high to control airway bacterial infections (MOI = 10^4^),^160^ which further supports the conclusion that phage penetration to the lungs has rather low efficiency.

“Backward penetration” of bacteriophages from the blood to the gastrointestinal tract is also possible. Systemically administered phages have been observed in feces of calves and mice,^117^, ^120^ in intestines of mice, rabbits and chickens^80^, ^149^, ^150^, ^161^ and in murine stomach.^89^, ^139^ Stomach penetration from blood was studied in a model where mice were or were not prevented to swallow. Phage titer in the stomach was substantially lower in non‐swallowing animals. Hence, the authors proposed that “reverse penetration” of phage occurred by salivary glands and other areas of the upper gastrointestinal tract, and possibly on mucosal surfaces of the respiratory tract.^89^ In phage penetration to the gut, a specific concern may relate to possible phage propagation on gut bacteria. If it happens, observed phage titers can falsely support a high rate of phage backward penetration. Geier et al,^120^ who investigated germ‐free animals, observed little if any phage lambda in feces after IM, IP, or IV injections. Since the dose was above 10^12^ PFU per mouse, the “backward penetration,” though possible, seems to be rather poor.

## 4 PHAGE CLEARANCE

### 4.1 Time of phage clearance from blood

Phage pharmacokinetics in the presence of sensitive bacteria is fundamentally different from that of chemical drugs due to phage replication, as has been demonstrated in extensive analyses and mathematical modeling.^16^, ^17^, ^18^ Hypothetically, in the absence of sensitive bacteria, the phage itself might behave as a conventional drug. This hypothesis is not easy to be verified due to scanty and incoherent experimental data on the pharmacokinetics of nonreplicating phages. Cerveny et al^152^ reported a phage half‐life in mice of 2.2 hours, while Molenaar et al^162^ and Tiwari et al^151^ reported 4.5 hours. Accumulating data from available relevant reports (Figure S1) revealed that a semilog plot of phage decrease against time was not linear, demonstrating that phage clearance slows down with time. For example, median phage half‐life was 1.3 hours when calculated from the second hour after administration, but 4.0 hours for the 6th and 7.9 hours for the 12th hour after administration (the equation for this calculation is presented in Figure S1). This may suggest that phage half‐life is dose dependent or it is significantly affected by phage particles already accumulated by filtering organs and cells.

The potential effect of phage morphology on the time of clearance is vague, but Hájek^128^ reported that T2 phage (*Myoviridae*, approx. 90 × 200 nm in size) was removed much faster than phiX174 phage (*Microviridae*, approx. 30 nm in diameter) from the circulation of newborn germfree pigs. This suggests that large phage virions could be easier to filter out in vivo than small ones.

Phage pharmacokinetics can be markedly changed by encapsulation of bacteriophages. Most of available data indicate prolonged release of phage, close to constant release and different to bolus administrations.^54^, ^55^, ^163^ In addition to delivering control release systems, encapsulation protects bacteriophages and allows for longer circulation in human or animal body. This can be both by protection from chemical stress and by making phage less visible to the immune system and less susceptible to neutralization.^54^, ^164^

### 4.2 Filtering organs

As discussed in a previous section (see section 3.2), liver and spleen are considered to be the main organs that filter out circulating phages.^120^, ^125^, ^127^, ^149^ Since they are elements of the MPS, settled by numerous phagocytes, they are further considered major tools of phage clearance from animal and human bodies and are actively engaged in phage neutralization. Comparing between spleen and liver, it is the spleen that usually accumulates the highest titers of phages^113^, ^120^, ^137^, ^149^, ^165^ and also that often contains active phages for the longest time, longer than blood or liver.^121^, ^151^, ^153^ It is unclear whether the high capacity of the spleen to accumulate phage particles correlates with efficient phage neutralization. Splenectomy in dogs had no appreciable effect on the phage clearance curve (although the phage blood titer was tested for 1.5 hours only).^137^ Further, Inchley,^127^ who investigated phage accumulation in tissues by detection of radiation from ^51^Cr‐labeled T4 phage (not by antibacterial activity of the phage), found that 70 to 90% of phage was concentrated in the liver. The phage was observed in both spleen and liver of investigated mice, but it decreased much faster in the liver. Thus, it is probable that both liver and spleen accumulate phage particles very effectively, but it is the liver that rapidly inactivates phages, specifically by Kupffer cells.

Renal clearance plays an important role in the removal of many drugs. It mainly reflects the excretion of a drug into the urine. Bacteriophages were found to be stable in human and animal urine in vitro^142^, ^155^, ^166^; in vivo, many researchers detected active phages in kidneys and/or urine, either in animals^113^, ^123^, ^137^, ^147^, ^149^ or in humans.^167^ Human studies were conducted during oral phage therapy in various types of infections. However, phages were not detected in the urine of all patients: in 87.3% (N = 55) of treated children^167^ and in 35% (N = 26) of treated adults.^168^ Most of the researchers who studied phage excretion in animal models observed high variability between individuals and irregular occurrence (not in all animals) of active phages in the urine, even in systemic deliveries. Urine titers were even a few orders of magnitude lower than blood titers,^113^, ^123^, ^137^, ^147^, ^149^ and kidneys were concluded as not able to concentrate bacteriophages to the extent observed in the liver and the spleen.^137^ This is in line with studies in quantum dot models, where large model particles (>8 nm) were not found in the bladder but instead were trapped by the MPS.^169^ This implies that the major feature directing phage particles to the MPS instead of renal filtration is the large size of phage particles. Still, phage secretion to urine is possible, but the process seems to be limited and dependent on experimental conditions.

A factor that seems to have the major effect is the dose. Schultz and Neva^141^ concluded a dose dependency in phage penetration to the bladder, and they proposed 10^9^ PFU as a minimum dose for phage detection in urine in IV injected mice. This is in line with dose dependency demonstrated in phage therapy of experimental urinary tract infection in mice.^155^ Studies in dogs showed that the rate of phage excretion to urine paralleled phage concentration in plasma. This relation was proposed by the authors as consistent with a process governed by diffusion,^137^ but to the best of this author's knowledge, no studies elucidating the exact mechanism of phage excretion by kidneys have been published. In systemic administrations, phage concentration in plasma is strongly related to dose. Pouillot et al^113^ observed the effect of administration route on phage penetration to urine in rats. IP‐administered phages entered the urine. However, when SC‐administered, the phages were not detectable in urine but found in many tissues. Possibly IP administration led to higher phage titer in blood than that achieved by SC administration, as previously demonstrated in mice.^124^

### 4.3 Immune response

Extensive reviews presenting bacteriophage interactions with the immune system have been published in recent years.^144^, ^170^, ^171^, ^172^ Here, an update and recapitulation of major immunological processes that govern phage pharmacokinetics will be proposed.

The immune system plays the key role in phage clearance from animal and human bodies. As discussed above (see Filtering organs section), renal loss of phages plays a marginal role in comparison to phage neutralization by elements of the filtering macrophage phagocytic system (MPS or RES). One should note that the immune system removes phage particles even when no specific response to bacteriophages has yet developed. This is due to the nonspecific mechanism of the innate immune response, capable of removing exogenous objects without specific recognition.

Innate immunity (nonspecific) is the first line response which affects every phage that has entered an animal or human body. In general, innate immunity is executed by a wide collection of leukocytes i.e., white blood cells. These are mainly phagocytes—macrophages and monocytes, neutrophils, tissue dendritic cells, and mast cells—but also eosinophils, basophils, and natural killer cells. Phagocytes circulate around the body, but they are also the key fraction of cells in large organs, such as spleen and liver, which have been demonstrated as major “phage traps” inside bodies (see section 4.2). Consistently, phagocytosis seems to be the major process of bacteriophage neutralization.^19^, ^120^, ^127^, ^146^ In addition to the direct effect of phage inactivation, phagocytosis is the first step in processing and presenting phage antigens by antigen presenting cells (APCs, mainly dendritic cells and macrophages). Thus, phagocytosis initiates pathways necessary to develop a specific immune response.

Humoral (noncellular) elements of innate immunity are represented mainly by the serum complement system, an enzymatic cascade that without specific recognition initiates removal of invading microorganisms.^173^ In the early studies of bacteriophages, the complement system was investigated and discussed as the properdin system,^138^ properdin being a serum glycoprotein that can both initiate and positively regulate an alternative complement pathway.^174^ Either heat inactivation or chemical (zymosan) inactivation of the complement system resulted in increased phage survival in animal sera in vitro.^19^, ^138^, ^175^ Prior injection of zymosan to animals resulted in increased (more than 1 order of magnitude) blood phage titer in comparison to untreated animals.^138^ This demonstrates that the complement system is engaged in phage neutralization in vivo. A natural capability of this system is to neutralize viruses directly by destroying viral particles, which is in line with the observations of in vitro neutralization of phage virions by naive sera. Complement also facilitates phagocytosis, playing the role of opsonizing agent, which further contributes to phage neutralization in vivo.^126^, ^128^, ^176^

Differences in phage susceptibility to neutralization by the complement system can be responsible for differences in phage ability to maintain activity in the animal circulation. So‐called “long‐circulating phages” are mutants capable of remaining active circulating in blood for longer than parental phages. They have been proposed as more effective in phage therapy. Typically, they are selected by repeated passages in animals’ circulation.^116^, ^177^ Sokoloff^178^ demonstrated by engineering phage particles that a long‐circulating phenotype in rat model could be generated by introducing peptides with C‐terminal lysine or arginine on phage capsid surfaces. This resulted in lower sensitivity of the phage to complement‐mediated neutralization. Interestingly, the mutation identified following in vivo selected long‐circulating phages was located in the gene coding major capsid protein and it resulted in substitution of glutamic acid for lysine.^179^ Although the authors proposed lower phage susceptibility to MPS, long circulating phenotype may instead be due to lower phage susceptibility to the complement system.

Innate immunity can be boosted by universal molecular patterns associated with pathogens (pathogen‐associated molecular patterns [PAMPs]) for example, bacterial DNA (CpG), peptidoglycan, or lipopolysaccharide. Boosting innate immunity basically means induction of inflammation, which substantially increases the capacity of the immune system to remove foreign objects. However, purified phages (in contrast to raw phage lysates) have never been demonstrated to expose PAMPs effectively. They were demonstrated as not able to induce an inflammatory response^180^ and even can have anti‐inflammatory effects on the immune system.^181^, ^182^ Thus, the phage virion itself probably acts in a way that facilitates its longer circulation in animal or human bodies, however this issue needs to be further explored.

Adaptive immunity (specific) is not a separate mode of response, but instead enhances and expands nonspecific, innate functions of the immune system, adding an ability to target the response and to be much more efficient. The first distinctive feature of adaptive immunity is the requirement that it must be “trained” to recognize and respond to foreign epitopes. The second is the fact that adaptive immunity is fundamental to immunological memory ie, retention of enhanced abilities to recognize and respond to foreign epitopes. Major effectors of the specific immune response are lymphocytes which are able to develop a variety of specific receptors (T and B cells), and antibodies which are potent elements of the specific humoral response as secreted by B cells.^183^ These specific elements cooperate with nonspecific elements, mainly by facilitating phagocytosis of antigens opsonized with antibodies or by activation of the complement system with antibodies that bind to foreign epitopes in the classical activation pathway.

Antibodies specific to phage virions have been by far the most frequently investigated and acknowledged part of mammalian immune reactions to phages. Their induction after phage administration has been observed in animal models^19^, ^38^, ^50^, ^100^, ^118^, ^175^, ^184^, ^185^, ^186^, ^187^ and in humans.^131^, ^188^, ^189^ Phage‐specific antibodies may also rise from natural contact with phages that are ubiquitous in the environment and phages in human or animal bodies which make up the natural phageome.^27^, ^188^, ^190^, ^191^, ^192^ Notably, phages are complex structures composed of many proteins. Therefore, antibodies induced by one phage may also react and change pharmacokinetics of another phage that exposes at least some similar epitopes.^175^

As can be anticipated, in most cases in vivo phage‐specific antibodies decreased concentration of active phages in circulation and ex vivo specific sera inactivated virions; thus antibodies can have a devastating effect on the bacteriophage.^19^, ^100^, ^129^, ^131^, ^188^, ^193^, ^194^ In some cases, however, expected antibody formation in humans was not observed^32^ or had no effect on phage ability to control bacterial infection.^195^ This is in line with the fact that an efficient specific immune response requires enough time and an appropriate dose. In rodents, coliphages given in drinking water or IP injected did not induce high levels of specific antibodies for at least 3 weeks.^47^, ^50^, ^196^ IP‐applied *Pseudomonas* phages against experimental infection in mice induced phage‐specific antibodies many days after the time‐frame of effective treatment.^197^ T4 phage applied orally to mice required high doses to induce antibodies that neutralized the phage.^50^ Notably, antibodies neutralize bacteriophage activity, but they do not cause immediate disappearance of phage particles from the circulation. This was demonstrated by quantitative polymerase chain reaction (qPCR) in mice preimmunized to T4 phage. A substantial timelapse between “microbiological disappearance” and true clearance of phage particles from the circulation was observed.^198^ Thus, adaptive immunity may contribute to phage clearance, but first, this is more in terms of inactivation than physical removal, and second, it only happens when conditions of previous and/or current exposure to bacteriophages allow for effective “training” of the immune system. Otherwise, it does not add to “default” activity of innate immunity.

## 5 CONCLUSIONS

**Figure 6:**
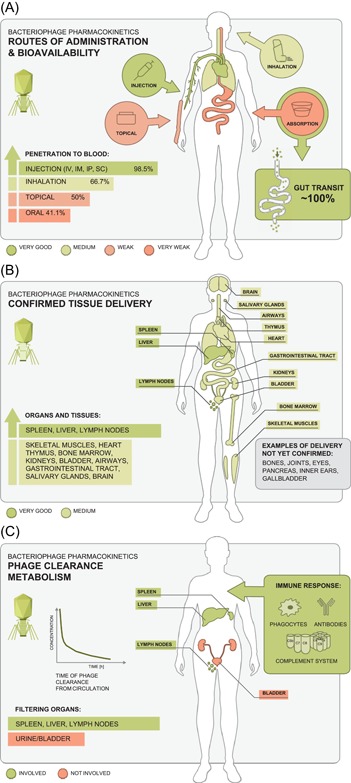
Routes of bacteriophage access to the body A, phage circulation in tissues B, and phage clearance in animals and humans C [Color figure can be viewed at wileyonlinelibrary.com]

Bacteriophage efficacy in entering animal or human bodies strongly depends on the route of administration. Oral administration effectively delivers phages to the gastrointestinal tract (Figure 6A). The efficiency of gut transit (fecal/cecal recovery of phage) is dose dependent. Differences were also observed between taxonomic groups of animals together with the strong significance of individuals’ age. The young individuals demonstrated higher phage recovery. This is probably due to differences between species and between young and older individuals in their gut physiology, including coexisting microbiomes and secretion of digestive compounds. However, stomach acidity neutralizers have not been revealed by the systematic analysis conducted herein as capable of significantly improve phage travel through the alimentary tract. This unexpected observation is contradictory to common expectations and it may result from a few reasons: (i) the sensitivity of phages to stomach pH is not a universal feature and many phages seem to be relatively acid‐resistant, (ii) there are fundamental differences between conditions in vivo and in vitro experiments where acidic sensitivity of phages has been typically assessed, like ionic composition and massive presence of organic macromolecules, and (iii) phage propagation in further sections of gastrointestinal tract may cover the effect of phage loss in the stomach. This is in line with the strong effect exerted by the presence of sensitive bacteria on phage fecal recovery. Phage shedding was improved either by infecting/artificially introduced bacteria or by natural symbionts. Phage replication in the gut seems to outweigh all other factors influencing the efficiency of phage recovery.

Although very good for delivering phage to alimentary tract, oral route of administration is the least effective for systemic penetration of bacteriophages. This suggests that phage absorption in the gastrointestinal tract is limited, but it evidently correlates to phage dose. The more phage is applied per os, the better chance for successful systemic dissemination. No effect of acidity neutralizers or stomach probes was found. Also, systemic penetration via the airways does not seem effective but is possible. The most efficient delivery can be achieved by injections (IV, IP, IM) that allow for effective phage dissemination within minutes. Thus, phage ability to disseminate in blood and tissues is strongly dependent on the route of administration (Figure 6A).

Once delivered systematically, bacteriophages may reach virtually all organs and tissues (Figure 6B): skeletal muscles, heart, thymus, bone marrow, kidneys, bladder, salivary glands with saliva, and even brain, although these organs do not appear to accumulate phages. Penetration to the lungs and “reverse penetration” to the lumen of the gastrointestinal tract are also possible but rather poor. There are still some organs and tissues important as therapeutic targets that have never been demonstrated as available for phage penetration, for example joints, bones, pancreas, eye, and others (Figure 6B).

Phage accumulation was observed in spleen, liver and lymph nodes as elements of the mononuclear phagocytic system representing the nonspecific part of the immune response. Phage clearance (Figure 6C) is mostly due to phagocytosis of phage particles in the liver and, to a lesser extent, in the spleen. Renal secretion has a marginal role, probably due to the large size of phage particles. Thus, the immune system plays a key role in phage clearance, even when no specific response to bacteriophages has yet developed. Phage titers in the blood shortly after intravenous delivery (up to 0.5 hours) are generally lower than could be calculated from simple dilution of the phage dose in the blood or body volume. This implies that capture of phage by the MPS is very rapid. Later, phage clearance slowed down, suggesting that phage half‐life (when no propagation occurs) is dose dependent or it is affected by phage particles already accumulated by filtering organs and cells.

## 6 STUDY LIMITATIONS

A systematic review, by definition, depends on the set of available data that determine the outcome of the analysis. Here, the review was aimed at evaluating phage penetration, circulation, and clearance in vertebrates. However, representation of particular taxonomic groups in available reports appeared to be extremely imbalanced. Specifically, most of the investigated animals are mammals, with high prevalence of the popular laboratory animal, mice. In birds, almost all studies were on chickens (with only one on quails). No experiments in reptiles or amphibians were found. Fish were poorly represented (14 experiments) and thus were analyzed together as one systematic group. Also, many administration routes or types of phages were poorly represented. These constitute gaps in the current state of research. Further, selected factors that potentially influence phage penetration and/or gut transit were analyzed. Others were not for example the use of antibiotics and other types of drugs, the specific composition of natural microbiomes, diet, and diseases other than infections by phage‐sensitive bacteria. Phage pharmacokinetics can be substantially changed by phage encapsulation, with a high variety of encapsulation systems that result in a multitude of chemically and physically different phage preparations (an extensive review of this topic has been recently presented by Malik et al^54^) Thus, other or unidentified factors that impact systemic penetration and gut transit of bacteriophages may still exist. As a consequence, the systematic analysis revealed (by statistics) overall trends, but these trends do not rule out exceptions that can be observed in specific experimental conditions. Many of these exceptions were cited herein as contrary to the overall trends. Both evident exceptions from identified trends and gaps in the available data point out areas for further investigations of phage penetration, circulation, and clearance in animal and human bodies.

## Supporting information

Supporting information
