# Supplementary material for: Phage therapy: What factors shape phage pharmacokinetics and bioavailability? Systematic and critical review
Source: Med Res Rev. 2019 Mar 19;39(5):2000–25. doi: 10.1002/med.21572 (PMC6767042; doi:10.1002/med.21572)
Supplement: Supplementary file 1 — Supporting information [file MED-39-2000-s001.docx]

**Supplementary material**

**Systematic review of publications on bacteriophage gut transit and on systemic penetration of bacteriophages in animals and humans**

**Aim** – to identify factors that facilitate or hinder phage ability to penetrate human and animal bodies, including phage transit through the gut

**Methods**

**Search strategy**: All PubMed records up to Dec 31st 2016 were used. The search was executed as follows: *((phage) OR bacteriophage) AND ((per os) OR (gut) OR (intestine) OR (fecal recovery) OR (route of administration) OR (oral) OR (inhalation) OR (intranasal) OR (intraperitoneal) OR (intramuscular) OR (subcutaneous) OR (intracranial) OR (intravenous) OR (parenteral) OR (experimental infection) OR (skin penetration) OR (wound) OR (transdermal) OR (penetration) OR (cutaneous) OR (circulation))*. All listed titles were analyzed for their topic. In the first round, reports of original work were analyzed directly. Since reviews did not present laboratory data, thus not meeting inclusion criteria, their lists of references were used to complete the analyzed list of titles. As a result, all titles within the scope of this review were selected for abstract analysis. Specifically, titles were selected when they indicated phage application into any vertebrate for any purpose. In the second round, abstracts were analyzed and reports within the scope of this review were selected for full text analysis. Specifically, if no exclusion criteria were evident from the abstract, the report was selected for further analysis. If the abstract was not available, the relevant report was also subjected to full text analysis. In the third round, full texts were analyzed and data were extracted. All selected full texts had to be accessed and analyzed.

**Article inclusion and exclusion criteria**

In order for an article to be included in this review (1) it must include studies in vertebrates, (2) it must include studies in living animals or humans, (3) the studied bacteriophage must be identified at least by its host specificity, (4) the studied bacteriophage must be natural, (5) the studied bacteriophage must have been detected directly, i.e. by its activity (indirect evidence such as positive therapeutic effects or immune response were excluded). Article exclusion criteria were reports or studies that (1) were not published in peer-reviewed journals (e.g. dissertations), (2) report studies of unspecified antibacterial preparations attributed to bacteriophages, (3) no living vertebrate participated in the study (ex vivo models, worm models, artificial systems and bioreactors, numerical simulations, etc), (4) the studied bacteriophage was modified with foreign elements, e.g. by phage display, encapsulation, chemical conjugation, etc. (unmodified vectors for phage display were included), (5) the bacteriophage was delivered via more than one route of application in the same experiment, (6) in the case of gut transit, an unspecified dose or recovery titer of the phage, (7) in the case of penetration, the phage was applied and detected in the same body site.

**Data extraction**

Data extraction was executed by the author of this review between 01.01.2017 and 31.05.2017 and double checked between 01.07.2017 and 30.09.2017. Reasons for data inclusion or exclusion were recorded. Data were extracted using a pre-defined form.

Phage penetration data were extracted using a form including (1) study authors’ names, (2) publication dates and citation, (3) bacteriophage specificity (bacterial host of the bacteriophage), (4) bacteriophage name, (5) bacteriophage type (taxonomic group), (6) type of the bacterial host (Gram-positive or Gram-negative), (7) route of phage administration, (8) species names of tested animals, (9) tested animals’ age (young or adult), (10) presence of bacteria sensitive to the studied phage (present or not), (11) N in the groups of tested animals, when not specified or unclear, was designated “1” on the assumption that at least one individual was investigated, when a range of N (e.g. 5–7) was given the lowest value was used, and when the same effect was investigated and confirmed at more than one time point N was summarized, (12) the purpose of the study (pharmacokinetic studies or phage therapy), (13) in the case of oral treatment, acidity neutralized (used or not used), (14) in the case of oral treatment, stomach probe or force feeding (used or not used), (15) in the case of oral treatment, dose, (16) result, (17) strength of the result (1–3, see below), (18) short description of the result, specifying where the phage was detected. Data collection included additional literature surveys, searches of databases or contacting authors of analyzed articles when any data were not given or not clear. Data from points (1) to (3) were obligatory and had to be available. Others were denoted as unspecified/unclear if they were not available. In case of any doubt, information was not included. Within categories of factors, e.g. (7) route of phage administration, types of factors were identified (e.g. intravenous, subcutaneous, oral, etc.).

Gut transfer of phage data were extracted using a form including (1) study authors’ names, (2) publication dates and citation, (3) bacteriophage specificity (bacterial host of the bacteriophage), (4) bacteriophage name, (5) bacteriophage type (taxonomic group), (6) type of the bacterial host (Gram-positive or Gram-negative), (7) species name of tested animals, (8) tested animals’ age (young or adult), (9) presence of bacteria sensitive to the studied phage (present or not) (10) N in the groups of tested animals, when not specified or unclear, was designated “1” on the assumption that at least one individual was investigated, and when a range of N (e.g. 5–7) was given the lowest value was used, (11) purpose of the study (pharmacokinetic studies or phage therapy), (12) acidity neutralized (used or not used), (13) stomach probe or force feeding (used or not used), (14) daily dose normalized per gram of body weight (for numerical calculation of the result, see below), (15) maximum phage recovery from fecal/cecal content as observed in the experiment (for numerical calculation of the result, see below), (16) strength of the result (1-3, see below), (17) short description of the result, specifying where the phage was detected. Data collection included additional literature surveys, searches of databases or contacting authors of analyzed articles when any data were not given or not clear, approximation of phage titer was accepted in the range +/- 20%. Inclusion of data from points (1) to (3) was obligatory. Other data were denoted as unspecified/unclear if they were not available. In case of any doubt, information was not included. Within categories of factors, types of factors were identified (e.g. in (3) bacteriophage specificity: *E. coli* phage, staphylococcal phage, etc.).

**Data transformation and statistics**

Due to the characteristics of available data, phage penetration results were defined as positive (phage detected in any organ or tissue, numerical: 1) or negative (phage not detected, numerical: 0). It was not possible to express the result as an observed phage titer due to the non-comparable character of experimental data, e.g. phage titer in brain v. phage titer in kidney. Additionally, strength of the result was defined as 1–3, according to the fraction of individuals in an experimental group that were positive for the phage: 1 for 0–50 % individuals, 2 for 51%–99% of individuals, and 3 when all individuals were positive for the phage. A second parameter for weighting the result was the number of studied animals per group (N).

Results that were expressed as 0–1 data were first used for analysis of phage penetration influencing factors by logistics regression. Data were further transformed as result x strength of the result and analyzed by linear regression (Figure 3). Factors (3) and from (5) to (10) were analyzed and defined according to p values: for p < 0.01 the factor was considered highly significant (red dots in Figure 1), for 0.01 <p <0.05 the factor was considered significant (yellow dots in Figure 1), and for p > 0.5 the factor was considered insignificant (grey dots in Figure 1). Factors selected as significant or highly significant were further analyzed by chi-square analysis with a weighting parameter (N x strength of the result). Chi-square analysis was used to identify significant differences between particular types of factors within factor categories (e.g. to compare penetration of *Podoviridae* to that of *Myoviridae*, or penetration after oral administration to that after intravenous injection). Correlation of phage dose in oral application and the effective/ineffective penetration of phage to tissues and organs was analyzed by Spearman's rank correlation.

The phage gut transit result was calculated from phage titers as A/B where: (A) daily dose normalized per gram of body weight (see also Table S5), and (B) maximum value of phage recovery from fecal/cecal content as observed in the experiment. Thus, the calculation represented the relative rate of increase/decrease of phage amount that was applied orally and passed through the gastrointestinal tract. Number of studied animals per group (N) was used for weighting the result. Due to the commonly very efficient transit in all individuals, fractions of positive individuals in groups had no applicability in this analysis.

Numerical values of the results (A/B) were transformed by log10 and used to analyze factors influencing phage gut transit by linear regression. Factors (3) and from (5) to (10) were analyzed and defined according to p values: for p < 0.01 the factor was considered highly significant (red dots in Figure 1), for 0.01 <p <0.05 the factor was considered significant (yellow dots in Figure 1), and for p > 0.5 the factor was considered insignificant (grey dots in Figure 1). Factors selected by linear regression as significant or highly significant were further analyzed by ANOVA (when there were more than two categories of a factor) or by Mann-Whitney U-test (when only two categories of a factor), with the use of weight parameter N and purpose of the study. ANOVA or Mann-Whitney U-test was used to identify significant differences between particular types of factors within factors.

**Results and Discussion**

**Data extraction statistics**

years of publications covered by the search: 1940–2016

number of titles in the first round of analysis (by title): 3279

number of titles selected for the second round of analysis (by abstract): 379

number of titles selected for the third round of analysis (by full text): 221

number of individual experiments that were extracted: 235

number of individual experiments extracted for phage penetration topic: 144

number of individual experiments extracted for phage gut transit topic: 91

**Extracted data**

Extracted data are listed in **Table S1** and **Table S2**.

**Analysis**

Data were analyzed as described in Methods. The major part of the results and conclusions is presented in the main text of this review. Here, additional aspects representing statistically significant effects (Figures 1, 2, and 3) not included in the main text will be presented.

Bacteriophage taxonomy, which is generally related to phage morphology, may potentially affect phage ability to penetrate animal and human bodies. As revealed by the systematic analysis herein: (i) no relation was found between phage morphology/taxonomy and the efficiency of phage transit, and (ii) phage morphology affected phage penetration into the system (p=0.0117 in a logistic regression model and p<0.0001 by Spearman correlation).

Small round bacteriophages seem to penetrate living systems more easily than those with extended tails or those of filamentous morphology: Podoviridae penetrated significantly better than Myoviridae (p<0.0001), Siphoviridae (p=0.0015) and Inoviridae (p<0.0001). Inoviridae, a group of long filamentous phages, penetrated worse than all other groups except Myoviridae (insignificant): Podoviridae (p<0.0001), Microviridae (p=0.01748) or Siphoviridae (p=0.00036). Comparison of Myoviridae (long non-flexible tail) to Siphoviridae (even longer but flexible tail) revealed significantly worse penetration of Myoviridae (p<0.0001). These results imply that phage penetration is impeded by long morphological structures (the whole virion morphology or a long tail), especially by those structures that are non-flexible (tails).

Taxonomy of the treated animal, which from a practical point of view means differences in physiology of different species, also had a significant effect on phage penetration (p=0.0069 in a logistic regression model and p<0.0001 by Spearman correlation). Specifically, phage penetration in mice was significantly better that that in humans (p<0.0001) or chickens (p<0.0001), while no statistically significant difference was observed between mice and rats (p=0.48836) or mice and rabbits (p=0.18469). These results demonstrate that in vivo models making use of popular laboratory animals may be insufficient for investigation of phage penetration in other distant species including humans.

**Table S1**. Gut transit of bacteriophages, data extracted within the systematic analysis. U – unspecified or unclear, Myo – *Myoviridae*, Sipho – *Siphoviridae*, Podo – *Podoviridae*, Micro – *Microviridae*, mix – mix of bacteriophages, **+** – phages specific to Gram-positive bacteria, **-** – phages specific to Gram-negative bacteria, Y – young individuals treated, A – adult individuals treated, YES – used/present, NO – not used/not present, daily dose normalized – daily dose recalculated per 1 gram of body weight (Table S5); **Result** was (i) described, (ii) calculated as a **relative phage recovery rate** from a normalized daily dose (A) and fecal recovery of phage (B) as A/B.

| **author** | **year** | **reference** | **phage** | **phage type** | **phage specificity** | **Gram-type** | **tested species** | **young/adult** | **acidity neutralizer** | **sensitive bacteria** | **Daily dose normalized [pfu/g] (A)** | **maximum fecal/cecal recovery [pfu/g] (B)** | **Result: relative phage recovery rate: (B/A)** | **result described** | **N** | **comments** |
| --- | --- | --- | --- | --- | --- | --- | --- | --- | --- | --- | --- | --- | --- | --- | --- | --- |
| Abdulamir  et al. | 2014 | Ann Clin Microbiol Antimicrob 13:39 | CEH-183 | U | Escherichia coli | **-** | rat | A | YES | YES | 4x10^5^ | 4.1x10^5^ | **1.025** | phage detected in feces | 1 | daily dose calculated by the author of this review from average daily water uptake in rats |
|  |  |  |  |  |  |  |  |  |  |  | 1x10^6^ | 5.4x10^5^ | **0.54** | phage detected in feces | 1 |  |
| Ahmadi  et al. | 2016 | Front Microbiol. 7:1253 | PSE | Sipho | Salmonella | **-** | quail | Y | NO | YES | 1x10^6^ | 1x10^5^ | **0.1** | phage detected in feces | 10 |  |
| Bardina  et al. | 2012 | Appl Environ Microbiol 78(18): 6600-7 | mix | mix | Salmonella | **-** | chicken | Y | NO | YES | 5x10^7^ | 7x10^5^ | **0.014** | phage detected in cecal content | 4 |  |
|  |  |  |  |  |  |  | mouse | A | NO | NO | 5x10^6^ | 1x10^3^ | **0.0002** | phage detected in cecal content | 10 |  |
|  |  |  |  |  |  |  |  |  |  |  | 5x10^8^ | 1x10^4^ | **0.00002** | phage detected in cecal content | 10 |  |
| Bruttin and Brussow | 2005 | Antimicrob Agents Chemother Jul;49(7):2874-8 | T4 | Myo | Escherichia coli | **-** | human | A | YES | NO | 1.3x10^2^ | 3x10^4^ | **230.76923** | phage detected in feces | 15 |  |
| Carvalho  et al. | 2016 | BMC Microbiol 10:232 | mix | Myo | Campylobacter | **-** | chicken | Y | YES | YES | 5x10^3^ | 2x10^5^ | **40** | phage detected in feces | 15 |  |
| Chibani-Chennoufi  et al. | 2004 | Antimicrob Agents Chemother. 48(7):2558-69 | JS4 | Myo | Escherichia coli | **-** | mouse | A | YES | NO | 2.5x10^5^ | 3x10^5^ | **1.2** | phage detected in feces | 1 | the same animals used sequentially for testing phages JS4, JSD.1, JSL.6, JS94.1. Fecal recovery estimated from a graph |
|  |  |  |  |  |  |  |  |  |  |  | 2.5x10^4^ | 7x10^5^ | **28** | phage detected in feces | 1 |  |
|  |  |  |  |  |  |  |  |  |  |  | 2.5x10^3^ | 5x10^3^ | **2** | phage detected in feces | 1 |  |
|  |  |  |  |  |  |  |  |  |  |  | 2.5x10^2^ | 1x10^3^ | **4** | phage detected in feces | 1 |  |
|  |  |  | JSD.1 | Myo | Escherichia coli | **-** | mouse | A | YES | NO | 2.5x10^5^ | 8x10^6^ | **32** | phage detected in feces | 1 |  |
|  |  |  |  |  |  |  |  |  |  |  | 2.5x10^4^ | 2x10^6^ | **80** | phage detected in feces | 1 |  |
|  |  |  |  |  |  |  |  |  |  |  | 2.5x10^3^ | 8x10^4^ | **32** | phage detected in feces | 1 |  |
|  |  |  |  |  |  |  |  |  |  |  | 2.5x10^2^ | 1x10^4^ | **40** | phage detected in feces | 1 |  |
|  |  |  | JSL.6 | Myo | Escherichia coli | **-** | mouse | A | YES | NO | 2.5x10^5^ | 1x10^6^ | **4** | phage detected in feces | 1 |  |
|  |  |  |  |  |  |  |  |  |  |  | 2.5x10^4^ | 2x10^6^ | **80** | phage detected in feces | 1 |  |
|  |  |  |  |  |  |  |  |  |  |  | 2.5x10^3^ | 5x10^4^ | **20** | phage detected in feces | 1 |  |
|  |  |  |  |  |  |  |  |  |  |  | 2.5x10^2^ | 1x10^3^ | **4** | phage detected in feces | 1 |  |
|  |  |  | JS94.1 | Myo | Escherichia coli | **-** | mouse | A | YES | NO | 2.5x10^5^ | 1x10^7^ | **40** | phage detected in feces | 1 |  |
|  |  |  |  |  |  |  |  |  |  |  | 2.5x10^4^ | 2x10^6^ | **80** | phage detected in feces | 1 |  |
|  |  |  |  |  |  |  |  |  |  |  | 2.5x10^3^ | 8x10^4^ | **32** | phage detected in feces | 1 |  |
|  |  |  |  |  |  |  |  |  |  |  | 2.5x10^2^ | 1x10^4^ | **40** | phage detected in feces | 1 |  |
|  |  |  | JS94.1 | Myo | Escherichia coli | **-** | mouse | A | YES | YES | 2.5x10^2^ | 1x10^10^ | **40000000** | phage detected in feces | 2 | axenic mice inoculated with a phage sensitive strain |
| Denou | 2009 | Virology 388(1):21-30 | Mix (T4-like) | Myo | Escherichia coli | **-** | mouse | A | YES | NO | 2.5x10^8^ | 1x10^6^ | **0.004** | phage detected in colon content | 5 | daily dose calculated by the author of this review from average daily water uptake in mice |
|  |  |  |  |  |  |  |  |  |  | YES | 2.5x10^8^ | 1x10^8^ | **0.4** | phage detected in colon content | 5 |  |
| Fiorentin  et al. | 2005 | Avian Pathol. 34(3):258-63 | mix | U | Salmonella | **-** | chicken | Y | NO | YES | 1.5x10^9^ | 1x10^6^ | **0.00066666** | phage detected in cecal content | 5 |  |
| Galtier et al. | 2016 | Environ Microbiol. 18(7):2237-45 | AL505_P1 | Sipho | Escherichia coli | **-** | mouse | A | NO | YES | 1x10^6^ | 5x10^9^ | **5000** | phage detected in feces | 2 |  |
|  |  |  | AL505_P2 | Myo | Escherichia coli | **-** | mouse | A | NO | YES | 1x10^6^ | 8x10^8^ | **800** | phage detected in feces | 2 |  |
|  |  |  | AL505_P3 | Podo | Escherichia coli | **-** | mouse | A | NO | YES | 1x10^6^ | 1x10^9^ | **1000** | phage detected in feces | 2 |  |
|  |  |  | mix | mix | Escherichia coli | **-** | mouse | A | NO | YES | 1x10^6^ | 6x10^9^ | **6000** | phage detected in feces | 2 |  |
|  |  |  |  |  |  |  |  |  |  |  | 3x10^6^ | 1x10^9^ | **333.3** | phage detected in feces | 5 |  |
|  |  |  |  |  |  |  |  |  |  |  | 3x10^4^ | 3x10^9^ | **100000** | phage detected in feces | 5 |  |
| Houghton and Fuller | 1980 | Appl Environ Microbiol. 39(5):1054-8 | SY1 | Sipho | Streptococcus | **+** | chicken | Y | NO | YES | 5x10^5^ | 5x10^8^ | **1000** | phage detected in cecal content | 3 | maximum phage recovery given as "no less than". Morphology: Siphoviridae was concluded by the author of this review from an EM picture presented in the manuscript |
| Hurley  et al. | 2008 | Avian Dis. 52(4):599-607 | SP6 | Podo | Salmonella | **-** | chicken | Y | NO | YES | 4.3x10^4^ | 1.4x10^6^ | **32.558139** | phage detected in feces | 10 |  |
|  |  |  |  |  |  |  |  |  |  | NO | 4.3x10^4^ | 0 | **EXCLUDED** | phage not detected in feces | 10 |  |
| Jaiswal et al. | 2013 | Microbes Infect 15(2):152-6 | mix | U | Vibrio cholerae | **-** | rabbit | A | NO | NO | 2.5x10^4^ | 6x10^6^ | **240** | phage detected in feces | 3 |  |
| Jamalludeen et al. | 2009 | Vet Microbiol 136(1-2):135-41 | mix | Myo | Escherichia coli | **-** | pig | Y | YES | YES | 3x10^5^ | 1x10^11^ | **333333.3333** | phage detected in feces | 10 | animals pretreated with florfenicol; trial 3 |
|  |  |  |  |  |  |  |  |  |  |  | 1.8x10^5^ | 1x10^11^ | **555555.5556** | phage detected in feces | 13 | animals pretreated with florfenicol; trial 3 |
| Kittler et al. | 2013 | Appl Environ Microbiol. 79(23):7525-33 | mix | Myo | Campylobacter | **-** | chicken | A | NO | YES | 1.1x10^4^ | 1x10^1^ | **0.000909** | poor recovery of phage from feces | 9 | trial 1 |
|  |  |  |  |  |  |  |  |  |  |  | 5.3x10^4^ | 1x10^2^ | **0.0018867** | poor recovery of phage from feces | 9 | trial 2 |
| Ma et al. | 2016 | Poult Sci. 95(12):2911-2920 | Felix 01 | Myo | Salmonella | **-** | chicken | Y | NO | NO | 3x10^7^ | 8x10^3^ | **0.000266667** | phage detected in feces | 6 |  |
| Majewska  et al. | 2015 | Viruses. 7(8):4783-9 | T4 | Myo | Escherichia coli | **-** | mouse | A | NO | NO | 1x10^9^ | 2x10^8^ | **0.2** | phage detected in feces | 7 |  |
| Maura and Debarbieux | 2012 | Bacteriophage 2(4):229-233 | CLB_P1 | Podo | Escherichia coli | **-** | mouse | A | NO | YES | 2.5x10^4^ | 3x10^6^ | **120** | phage detected in feces | 4 | phage daily dose calculated from phage concentration in drinking water and average water uptake in mice, phage recovery approximated from graph |
|  |  |  | CLB_P2 | Myo | Escherichia coli | **-** | mouse | A | NO | YES | 2.5x10^4^ | 2x10^10^ | **800000** | phage detected in feces | 4 |  |
|  |  |  | CLB_P3 | Sipho | Escherichia coli | **-** | mouse | A | NO | YES | 2.5x10^4^ | 1.5x10^7^ | **600** | phage detected in feces | 4 |  |
| Maura and Debarbieux | 2012 | Antimicrob Agents Chemother 56(12):6235-42 | mix | mix | Escherichia coli | **-** | mouse | A | NO | YES | 7.5x10^7^ | 1x10^10^ | **133.3333333** | phage detected in feces | 4 | phage daily dose calculated from phage concentration in drinking water and average water uptake in mice, phage recovery approximated from graph |
|  |  |  |  |  |  |  |  |  |  |  | 6x10^9^ | 1.5x10^10^ | **2.5** | phage detected in feces | 4 |  |
| Maura et al. | 2012 | Environ Microbiol 14(8):1844-54 | mix | mix | Escherichia coli | **-** | mouse | A | NO | YES | 7.5x10^7^ | 2x10^9^ | **26.66666667** | phage detected in feces | 4 |  |
|  |  |  |  |  |  |  |  |  |  | NO | 2.5x10^8^ | 2x10^6^ | **0.008** | phage detected in feces | 2 |  |
| Reynaud  et al. | 1992 | Vet Microbiol 30(2-3):203-12 | CF0103 | Podo | Escherichia coli | **-** | rabbit | A | NO | NO | 1.3x10^6^ | 3,2x10^3^ | **0.0024615** | phage detected in cecal content | 2 |  |
|  |  |  |  |  |  |  |  |  |  | YES | 1.3x10^6^ | 3,2x10^10^ | **24615.38462** | phage detected in cecal content | 5 |  |
| Rozema et al. | 2009 | J Food Prot. 72(2):241-50 | mix | U | Escherichia coli | **-** | calf | Y | YES | YES | 4.7x10^6^ | 2x10^6^ | **0.4255319** | phage detected in feces | 4 |  |
| Sarker et al. | 2016 | EBioMedicine 4:124-37 | Mix (T4-like) | Myo | Escherichia coli | **-** | human | Y | YES | U | 3.3x10^1^ | 1x10^6^ | **303030.3** | phage detected in feces | 52 | https://clinicaltrials.gov/ct2/show/NCT00937274  Patients with acute diarrhea, not specified if infected with sensitive bacteria |
| Sarker et al. | (2016)  2017 | Environ Microbiol 2017 19(1):237-250, Epub 2016 Nov 13. | mix (T4 like, Nestle Phage Collection) | Myo | Escherichia coli | **-** | human | Y | NO | NO | 3.3x10^3^ | 1x10^3^ | **0.303030303** | phage detected in feces | 15 | healthy children, median age 7 |
|  |  |  | mix (Microgen ColiProteus) | mix | Escherichia coli, Proteus | **-** | human | Y | NO | NO | 3.3x10^4^ | 1x10^4^ | **0.303030303** | phage detected in feces | 10 | healthy children, median age 7 |
| Sarker et al. | 2012 | Virology 434(2):222-32 | Mix (T4 like, Nestle Phage Collection) | Myo | Escherichia coli | **-** | human | A | YES | NO | 1.3x10^4^ | 2x10^2^ | **0.0153846** | phage detected in feces | 42 |  |
| Sheng et al. | 2006 | Appl Environ Microbiol. 72(8):5359-66 | KH1 | U | Escherichia coli | **-** | sheep | A | NO | YES | 1x10^6^ | 1x10^6^ | **1** | phage detected irregularly in feces | 3 |  |
|  |  |  | SH1 | U | Escherichia coli | **-** | mouse | A | NO | YES | 5x10^8^ | 1x10^6^ | **0.002** | phage detected in feces | 3 |  |
|  |  |  | SH1, KH1 | U | Escherichia coli | **-** | mouse | A | NO | YES | 5x10^8^ | 1x10^6^ | **0.002** | phage detected in feces | 3 |  |
| Smith and Huggins | 1983 | J Gen Microbiol. 129(8):2659-75. | B44/1 | U | Escherichia coli | **-** | calf | Y | NO | YES | 1.4x10^6^ | 1x10^8^ | **71.4** | phage detected in feces | 7 | re-isolation from colon content |
|  |  |  |  |  |  |  |  |  |  |  |  | 2x10^5^ | **0.142857143** | phage detected in cecal content | 1 | table 4 of the original text, colostrum deprived calves |
|  |  |  |  |  |  |  |  |  |  |  |  | 1x10^9^ | **714.3** | phage detected in cecal content | 1 | table 4 of the original text, colostrum deprived calves |
|  |  |  |  |  |  |  |  |  |  |  |  | 5x10^11^ | **357142.8571** | phage detected in cecal content | 11 | table 6 of the original text, phage applied together with B44/1 |
|  |  |  |  |  |  |  |  |  |  |  |  | 2x10^10^ | **14285.71429** | phage detected in cecal content | 6 | table 5 of the original text, phage applied together with B44/2 |
|  |  |  |  |  |  |  |  |  |  |  |  | 1x10^7^ | **7.142857143** | phage detected in feces | 5 | table 7 of the original text, phage applied together with B44/3 |
|  |  |  | B44/2 | U | Escherichia coli | **-** | calf | Y | NO | YES | 1.4x10^6^ | 1x10^7^ | **7.1** | phage detected in feces | 7 | re-isolation from colon content |
|  |  |  |  |  |  |  |  |  |  |  |  | 3x10^7^ | **21.4** | phage detected in cecal content | 1 | table 4 of the original text, colostrum deprived calves |
|  |  |  |  |  |  |  |  |  |  | NO | 1.4x10^6^ | 4x10^5^ | **0.285714286** | phage detected in cecal content | 1 | table 4 of the original text, colostrum deprived calves |
|  |  |  | B44/3 | U | Escherichia coli | **-** | calf | Y | NO | YES | 1.4x10^6^ | 1x10^5^ | **0.071428571** | phage detected in cecal content | 1 | table 4 of the original text, phage applied together with B44/1, colostrum deprived calves |
|  |  |  |  |  |  |  |  |  |  |  |  | 5x10^10^ | **35714.28571** | phage detected in cecal content | 11 | table 6 of the original text, phage applied together with B44/1 |
|  |  |  |  |  |  |  |  |  |  |  |  | 5x10^7^ | **35.71428571** | phage detected in feces | 5 | table 7 of the original text, phage applied together with B44/1 |
|  |  |  | B44/1, B44/2 | U | Escherichia coli | **-** | calf | Y | NO | YES | 1.4x10^6^ | 3x10^8^ | **214.3** | phage detected in cecal content | 2 | table 4 of the original text, colostrum deprived calves |
|  |  |  | P433/1 | U | Escherichia coli | **-** | pig | Y | NO | YES | 1x10^6^ | 2x10^9^ | **2000** | phage detected in feces | 7 | table 12 of the original text, phage applied together with P433/2 |
|  |  |  |  |  |  |  |  |  |  |  | 1x10^5^ | 5x10^8^ | **5000** | phage detected in colon | 1 | table 13 of the original text |
|  |  |  | P433/2 | U | Escherichia coli | **-** | pig | Y | NO | YES | 1x10^6^ | 2x10^8^ | **200** | phage detected in feces | 7 | table 12 of the original text, phage applied together with P433/1 |
|  |  |  |  |  |  |  |  |  |  |  | 1x10^5^ | 2x10^4^ | **0.2** | phage detected in colon | 1 | table 13 of the original text |
|  |  |  | S13 | Micro | Escherichia coli | **-** | sheep | Y | NO | YES | 1x10^5^ | 1x10^9^ | **10000** | phage detected in colon | 1 | table 15 of the original text |
| Smith and Huggins | 1987 | J Gen Microbiol 133(5):1111-26 | B41/1 | U | Escherichia coli | **-** | calf | Y | NO | YES | 1.4x10^0^ | 1x10^10^ | **7142857143** | phage detected in feces | 7 |  |
|  |  |  | B117/1 | U | Escherichia coli | **-** | calf | Y | NO | YES | 1.4x10^-3^ | 4x10^8^ | **2.85714E+11** | phage detected in feces | 7 |  |
| Tanji et al. | 2005 | J Biosci Bioeng. 100(3):280-7 | SP15, SP21, SP22 | U | Escherichia coli | **-** | mouse | A | YES | YES | 5x10^8^ | 5x10^5^ | **0.001** | phage detected in feces | 6 |  |
|  |  |  |  |  |  |  |  |  |  |  | 5x10^6^ | 2x10^4^ | **0.004** | phage detected in feces | 6 |  |
| Weiss et al. | 2009 | Virology 393(1):16-23 | T4-like phages RB49 and RB69 | Myo | Escherichia coli | **-** | mouse | A | YES | YES | 2.5x10^3^ | 5x10^4^ | **20** | phage detected in colon | 3 | N represents K-12-colonized mice tested 4 h after inoculation with phage, phage titer approximated |
|  |  |  | T4 | Myo | Escherichia coli | **-** | mouse | A | YES | NO | 5x10^5^ | 6x10^5^ | **1.2** | phage detected in feces | 6 | germ-free mice |
|  |  |  |  |  |  |  |  |  |  | YES | 5x10^5^ | 1x10^8^ | **200** | phage detected in feces | 5 |  |
|  |  |  | ED6 | Myo | Escherichia coli | **-** | mouse | A | YES | NO | 5x10^4^ | 7x10^5^ | **14** | phage detected in feces | 5 | germ-free mice; phage concentration in feces approximated from a figure |
|  |  |  |  |  |  |  |  |  |  | YES | 5x10^4^ | 7x10^7^ | **1400** | phage detected in feces | 5 | phage concentration in feces approximated from a figure |
|  |  |  | T7 | Podo | Escherichia coli | **-** | mouse | A | YES | NO | 5x10^4^ | 2x10^5^ | **4** | phage detected in feces | 5 | germ-free mice; phage concentration in feces approximated from a figure |
| Wong et al. | 2014 | Int J Food Microbiol. 172:92-101 | Φst1 | Sipho | Salmonella | **-** | chicken | Y | NO | YES | 1.3x10^9^ | 2.1x10^9^ | **1.6** | phage present in cecal content | 6 | re-isolation from cecal content |
|  |  |  |  |  |  |  |  |  |  | NO | 1.3x10^9^ | 6.6x10^8^ | **0.5076923** | phage present in cecal content | 6 | re-isolation from cecal content |

**Table S2**. Penetration of bacteriophages into animals and human bodies, data extracted within the systematic analysis. U – unspecified or unclear, N – not applicable, Myo – *Myoviridae*, Sipho – *Siphoviridae*, Podo – *Podoviridae*, Ino – *Inoviridae*, Micro – *Microviridae*, mix – mix of bacteriophages, **+** – phages specific to Gram-positive bacteria, **-** – phages specific to Gram-negative bacteria, IP– intraperitoneal, IV – intravenous, IM – intramuscular, SC – subcutaneous, t – transdermal, et – endotracheal, inh – inhalation, vag – vaginal, i.nas. – intranasal, IU – intrauterine, rect. – rectal, Y – young individuals treated, A – adult individuals treated, YES – used/present, NO – not used/not present. **Result** was (i) described, (ii) denoted as 0 – phage penetration not observed or 1 – phage penetration observed, (iii) assessed for its strength as follow: 1 – effect observed in minority of individuals, 2 – effect observed in majority of individuals, 3 – effect observed in all animals.

| **author** | **year** | **reference** | **phage** | **phage type** | **Phage specificity** | **Gram-type** | **route of administration** | **tested species/ group** | **young/adult** | **In case of oral administration** | | | **sensitive bacteria** | **result described** | **result (0/1)** | **strength of the result** | **N in experimental groups** | **Comments (optional)** |
| --- | --- | --- | --- | --- | --- | --- | --- | --- | --- | --- | --- | --- | --- | --- | --- | --- | --- | --- |
|  |  |  |  |  |  |  |  |  |  | **dose (log10)** | **acidity neutralizer** | **stomach probe** |  |  |  |  |  |  |
| Barrow et al. | 1998 | Clin Diagn Lab Immunol 5(3):294-8 | R | U | Escherichia coli | **-** | IM | chicken | Y | N | N | N | YES | phage detected in blood, spleen, and brain | 1 | 3 | 1 |  |
|  |  |  |  |  |  |  |  | cow | Y | N | N | N | YES | phage detected in blood and in feces | 1 | 3 | 4 |  |
| Bartell et al. | 1963 | J Exp Med. 118:13-26 | 81 | U | Staphylococcus aureus | **+** | IP | mouse | A | N | N | N | NO | phage detected in plasma | 1 | 3 | 5 |  |
|  |  |  |  |  |  |  |  |  |  |  |  |  | YES | phage detected in plasma | 1 | 3 | 5 |  |
| Bartell et al. | 1965 | Nature. 1965 Jan 30;205:474-5 | 81 | U | Staphylococcus aureus | **+** | IP | mouse | A | N | N | N | NO | phage detected in plasma | 1 | 3 | 5 |  |
|  |  |  |  |  |  |  |  |  |  |  |  |  | YES | phage detected in plasma | 1 | 3 | 5 |  |
| Bennet and Foster | 1966 | J Dairy Sci 49(11):1350-6 | U | U | Streptococcus | **+** | t | mouse | A | N | N | N | NO | phage detected in blood in some mice | 1 | 1 | 30 |  |
| Borie et al. | 2008 | Avian Dis. 52(1):64-7 | BP1, BP2, BP3 (mix) | U | Salmonella | **-** | oral | chicken | Y | 8 | NO | NO | NO | phage infrequently detected in spleen, liver and heart | 1 | 1 | 22 |  |
|  |  |  |  |  |  |  |  |  |  |  |  |  | YES | phage infrequently detected in spleen, liver and heart | 1 | 1 | 22 |  |
| Borie et al. | 2009 | Avian Dis. 53(2):250-4 | BP1, BP2, BP3 (mix) | U | Salmonella | **-** | inh | chicken | Y | N | N | NO | NO | phage infrequently detected in spleen and/or liver | 1 | 1 | 33 | organs analyzed  as a pool |
|  |  |  |  |  |  |  |  |  |  |  |  |  | YES | phage infrequently detected in spleen and/or liver | 1 | 1 | 33 | organs analyzed as a pool |
| Bradley et al. | 1963 | Proc Soc Exp Biol Med. 113:686-8 | MSP8 | Sipho. | Actinomycetes | **+** | oral | mouse | A | 10 | NO | NO | NO | phage detected in blood | 1 | 3 | 27 |  |
| Bruttin and Brussow | 2005 | Antimicrob Agents Chemother 49(7):2874-8 | T4 | Myo. | Escherichia coli | **-** | oral | human | A | 7 | YES | NO | NO | phage not detected in plasma | 0 | 3 | 15 |  |
| Capparelli  et al. | 2010 | J Infect Dis. 201(1):52-61 | phi1 | U | Salmonella | **-** | IV | mouse | A | N | N | N | YES | phage detected in blood, liver and in gastrointestinal tract | 1 | 3 | 10 |  |
| Capparelli  et al. | 2007 | Antimicrob Agents Chemother. 51:2765-73 | M^Sa^ | U | Staphylococcus aureus | **+** | SC | mouse | A | N | N | N | YES | phage detected in blood, spleen, heart and kidneys | 1 | 3 | 5 | phage selected as the long-circulating strain |
| Carmody  et al. | 2010 | J Infect Dis. 201(2):264-71 | BcepIL02 | Podo | Burkholderia | **-** | IP | mouse | A | N | N | N | YES | phage detected in lungs | 1 | 3 | 9 |  |
| Cerveny  et al. | 2002 | Infect Immun. 70:6251-62 | 153A-5 | U | Vibrio vulnificus | **-** | IV | mouse | A | N | N | N | NO | phage detected in blood, liver and peritoneum | 1 | 3 | 1 | mice treated with iron dextran, N non specified |
| Chhibber  et al. | 2008 | J Med Microbiol 1508-13 | SS | Podo | Klebsiella pneumoniae | **-** | IP | mouse | A | N | N | N | NO | phage detected in blood and lungs | 1 | 3 | 1 |  |
| Chibani-Chennoufi  et al. | 2004 | Antimicrob Agents Chemother. 48(7):2558-69 | mix (T4-like) | Myo | Escherichia coli | **-** | oral | mouse | A | 9 | NO | YES | YES | no phage detected in liver or mesenteric lymph nodes | 0 | 3 | 4 |  |
|  |  |  |  |  |  |  |  |  |  | 7 | NO | NO | YES | no phage detected in liver or mesenteric lymph nodes | 0 | 3 | 4 |  |
| Christiansen et al. | 2014 | Appl Environ Microbiol. 80(24):7683-93 | FpV-9 | Sipho | Flavobacterium psychrophilum | **-** | oral | fish | Y | 8 | NO | YES | NO | phage detected in majority of animals in spleen, brain and kidneys | 1 | 2 | 24 |  |
| Corbel and Morris | 1980 | Br Vet J 136(3):278-89 | Tb | Podo | Brucella | **-** | IV | guinea pig | A | N | N | N | YES | phage detected in blood | 1 | 2 | 11 | phage morphology concluded by the author of this review basing on Morris et al. 1973, J. gen. Virol. 20 |
| Dąbrowska et al. | 2007 | Arch Microbiol. 187:489-98 | T4 | Myo | Escherichia coli | **-** | IP | mouse | A | N | N | N | NO | phage detected in blood, spleen, liver, muscle and subcutaneous melanoma tumor | 1 | 3 | 6 | mice bearing subcutaneous tumors of B16 melanoma |
| Denou | 2009 | Virology 388(1):21-30 | mix  (T4-like) | Myo | Escherichia coli | **-** | oral | mouse | A | 9 | YES | NO | NO | phage not detected in blood, spleen and liver | 0 | 3 | 5 |  |
|  |  |  |  |  |  |  |  |  |  |  |  |  | YES | phage not detected in blood, spleen and liver | 0 | 3 | 5 |  |
| Dor-On and Solomon | 2015 | Front Microbiol. 6:530 | M13KO7 | Ino | Escherichia coli | **-** | i.nas. | mouse | A | N | N | N | NO | phage detected in brain | 1 | 3 | 3 | M13KO7 phage is an empty vector derived from M13 phage; no other tissues tested |
| Dubos et al. | 1943 | J Exp Med. 78(3):161-8 | U | U | Shigella | **-** | IP | mouse | A | N | N | N | NO | phage detected in blood and brain | 1 | 2 | 8 | N summarized from two time points within 48 hours, higher phage dose (exp. 2) |
|  |  |  |  |  |  |  |  |  |  |  |  |  | YES | phage detected in blood and brain | 1 | 3 | 8 | N summarized from two time points within 48 hours, higher phage dose (exp. 2) |
| Duerr et al. | 2004 | J Virol Methods 116(2):177-80 | M13KO7 | Ino | Escherichia coli | **-** | oral | rat | A | 12 | NO | YES | NO | phage not detected in spleen | 0 | 3 | 2 | M13KO7 phage is an empty vector derived from M13 phage; no other tissues tested |
| Frenkel and Solomon | 2002 | Proc Natl Acad Sci U S A. 99:5675-9 | f88 | Ino | Escherichia coli | **-** | i.nas. | mouse | A | N | N | N | NO | phage detected by immunofluorescence in brain | 1 | 3 | 10 | f88 phage is an empty vector derived from fd phage; no other tissues tested |
| Geier et al. | 1973 | Nature 246:221-3 | lambda | Sipho | Escherichia coli | **-** | IM | mouse | A | N | N | N | NO | phage detected in blood, spleen, liver, kidney, thymus, and peritoneum | 1 | 3 | 1 | N unspecified |
|  |  |  |  |  |  |  | IP | mouse | A | N | N | N | NO | phage detected in blood, spleen, liver, kidney, thymus, and peritoneum | 1 | 3 | 1 | N unspecified |
|  |  |  |  |  |  |  | IV | mouse | A | N | N | N | NO | phage detected in blood, spleen, liver, kidney, thymus, and peritoneum | 1 | 3 | 1 | N unspecified |
|  |  |  |  |  |  |  | oral | mouse | A | 12 | NO | YES | NO | phage detected in blood, spleen, liver, kidney, thymus, and peritoneum | 1 | 3 | 1 | N unspecified |
| Georgakopoulos | 1968 | Arch Gynakol. 205(3):211-8 | T3 | Podo | Escherichia coli | **-** | IU | mouse | A | N | N | N | NO | phage detected in blood | 1 | 2 | 10 |  |
|  |  |  |  |  |  |  | vag | mouse | A | N | N | N | NO | phage detected in blood in majority of mice | 1 | 2 | 10 |  |
| Guang-Han et al. | 2016 | PLoS One. 11(7):e0158213 | C34 | Myo | Burkholderia | **-** | IP | mouse | A | N | N | N | NO | phage detected in spleen | 1 | 2 | 6 |  |
|  |  |  |  |  |  |  |  |  |  |  |  |  | YES | phage detected in spleen, liver and lung | 1 | 2 | 6 |  |
| Hájek | 1970 | Folia Microbiol (Praha). 15(2):125-8 | phiX174 | Micro | Escherichia coli | **-** | IV | pig | Y | N | N | N | NO | phage detected in blood | 1 | 3 | 3 | intracardial injection |
|  |  |  | T2 | Myo | Escherichia coli | **-** | IV | pig | Y | N | N | N | NO | phage detected in blood | 1 | 3 | 3 | intracardial injection |
| Hildebrand and Wolochow | 1962 | Proc Soc Exp Biol Med 109:183-5 | T1 | Sipho | Escherichia coli | **-** | oral | rat | A | 9 | NO | YES | NO | phage detected in lymph collected from cisterna chyli | 1 | 2 | 15 |  |
| Hodyra-Stefaniak  et al. | 2015 | Sci Rep.5:14802 | F8 | Myo | Pseudomonas aeruginosa | **-** | IP | mouse | A | N | N | N | NO | phage detected in blood, spleen, liver, kidneys, lymph nodes and muscles | 1 | 3 | 7 | only non-immunized mice data extracted to the analysis |
| Hoffmann | 1965 | Zentralbl Bakteriol Orig. 198(4):371-90 | T3 | Podo | Escherichia coli | **-** | et | mouse | A | N | N | N | NO | phage detected in blood, spleen, liver, kidney and salivary gland | 1 | 3 | 1 | N was not clearly specified |
|  |  |  |  |  |  |  | IM | mouse | A | N | N | N | NO | phage detected in blood, spleen, liver and salivary gland | 1 | 3 | 10 |  |
|  |  |  |  |  |  |  | oral | mouse | A | 8 | NO | YES | NO | phage detected in blood, spleen and liver | 1 | 2 | 72 |  |
|  |  |  |  |  |  |  | rect. | mouse | A | N | N | N | NO | phage detected in blood, spleen, liver and salivary gland | 1 | 3 | 10 |  |
| Huff et al. | 2003 | Poult Sci. 82(7):1108-12 | DAF6, SPR02 (mix) | U | Escherichia coli | **-** | inh | chicken | Y | N | N | N | NO | phage infrequently detected in blood in a low titer | 1 | 1 | 5 |  |
|  |  |  |  |  |  |  | IM | chicken | Y | N | N | N | NO | phage detected in blood | 1 | 3 | 5 |  |
| Inchley | 1969 | Clin Exp Immunol 5(1):173-87 | T4 | Myo | Escherichia coli | **-** | IV | mouse | A | N | N | N | NO | phage detected in blood, spleen and liver | 1 | 3 | 6 | N summarized from two experimental groups (the first experiment) |
| Jaiswal et al. | 2014 | Int J Med Microbiol. 304(3-4):422-30 | Mix (ATCC 51352-B1, B2, B3, B4, B5) | U | Vibrio cholerae | **-** | oral | mouse | A | 7 | NO | YES | NO | phage detected in spleen and liver | 1 | 3 | 24 | N represents four different time-points, six mice each |
| Jun et al. | 2014 | J Infect Dis. J Infect Dis.  210(1):72-8 | pVp-1 | Sipho | Vibrio parahaemolyticus | **-** | oral | mouse | A | 8 | NO | YES | NO | phage detected in blood | 1 | 3 | 3 |  |
| Keller | 1958 | Science 128(3326):718-9 | U | Sipho | Bacillus megaterium | **+** | t | mouse | A | N | N | N | NO | phage detected in blood in majority of mice | 1 | 2 | 32 | phage type was concluded by the author of this review from a pool of Keller's publications |
| Keller and Engley | 1958 | Proc Soc Exp Biol Med. 98(3):577-80 | U | Sipho | Bacillus megaterium | **+** | IP | mouse | A | N | N | N | NO | phage detected in blood, spleen, liver, kidney, lung, brain and intestine | 1 | 3 | 1 | phage type concluded by the author of this review from morphological description of the phage |
|  |  |  |  |  |  |  | oral | mouse | A | 9 | NO | YES | NO | phage detected in blood | 1 | 2 | 1 | phage type concluded by the author of this review from morphological description of the phage; N given by Keller and Engley as a cumulative number, but no information on N in groups (unspecified=1) |
| Kim et al. | 2008 | Microb Biotechnol. 1(3):247-57 | A511 | Myo | Listeria | **+** | IV | mouse | A | N | N | N | NO | phage detected in blood | 1 | 3 | 8 |  |
|  |  |  | Felix-O1 | Myo | Salmonella | **-** | IV | mouse | A | N | N | N | NO | phage detected in blood | 1 | 3 | 8 |  |
| Letarova  et al. | 2012 | Folia Microbiol (Praha). 57(1):81-3 | U | U | Escherichia coli | **-** | oral | horse | A | U | NO | NO | YES | phage not detected in blood | 0 | 3 | 3 | natural bacteriophages were detected in gut in approximate concentration 10^3^ and then detection in blood was conducted |
| Liu et al. | 2016 | J Aerosol Med Pulm Drug Deliv. 29(5):393-405 | D29 | Sipho | Mycobacterium | **N** | et | mouse | A | N | N | N | NO | phage detected in spleen, kidney and brain | 1 | 2 | 15 | phage detected also in esophagus, thus gastrointestinal absorption of the phage also possible |
|  |  |  |  |  |  |  | IP | mouse | A | N | N | N | NO | phage detected in spleen, kidney and brain | 1 | 3 | 15 |  |
| Madsen et al. | 2013 | Appl Environ Microbiol 79(16):4853-61 | FpV-9 | Sipho | Flavobacterium psychrophilum | **-** | IP | fish | Y | N | N | N | NO | phage detected in spleen, brain and kidneys | 1 | 3 | 12 | N: summary of reported effective time points |
|  |  |  |  |  |  |  |  |  |  |  |  |  | YES | phage detected in spleen, brain and kidneys | 1 | 3 | 20 | N: summary of reported effective time points |
| Malik and Chhibber | 2009 | J Microbiol Immunol Infect. 42(2):134-40 | Kphi1 | Podo | Klebsiella pneumoniae | **-** | IP | mouse | A | N | N | N | NO | phage detected in blood and lungs | 1 | 3 | 8 |  |
| Matsuzaki  et al. | 2003 | Infect Dis 187(4):613-24 | phiMR11 | Sipho | Staphylococcus aureus | **+** | IP | mouse | A | N | N | N | NO | phage detected in blood | 1 | 3 | 1 |  |
|  |  |  |  |  |  |  |  |  |  |  |  |  | YES | phage detected in blood | 1 | 3 | 1 |  |
| McCallin  et al. | 2013 | Virology. 2013 Sep 1;443(2):187-96 | mix (Microgen ColiProteus cocktail) | mix | Escherichia coli, Proteus | **-** | oral | human | A | 8 | YES | NO | NO | no phage detected in the blood | 0 | 3 | 5 | substantial contribution of T7-like phages, further: T4-like phages |
|  |  |  |  |  |  |  |  |  | Y | 8 | YES | NO | NO | no phage detected in the blood | 0 | 3 | 10 | substantial contribution of T7-like phages, further: T4-like phages |
| McVay et al. | 2007 | Antimicrob Agents Chemother. 51(6):1934-8 | Pa1, Pa2, Pa11 (mix) | mix | Pseudomonas aeruginosa | **-** | IM | mouse | A | N | N | N | NO | phage detected in blood, spleen and liver | 1 | 3 | 3 |  |
|  |  |  |  |  |  |  | IP | mouse | A | N | N | N | NO | phage detected in blood, spleen and liver | 1 | 3 | 3 |  |
|  |  |  |  |  |  |  | SC | mouse | A | N | N | N | NO | phage detected in blood, spleen and liver | 1 | 3 | 3 |  |
| Morton and Perez-Otero | 1945 | J Bacteriol. 49(3):237-44 | U | U | Shigella | **-** | IP | mouse | A | N | N | N | YES | phage detected in blood | 1 | 3 | 3 |  |
| Muir and Blakemore | 1960 | Surg Forum. 10:339-42 | U | U | Staphylococcus aureus | **+** | IP | mouse | Y | N | N | N | NO | phage detected in spleen | 1 | 3 | 21 | N summarized from individual time-points (N=3 each) |
|  |  |  |  |  |  |  |  |  |  |  |  |  | YES | phage detected in spleen | 1 | 3 | 21 | N summarized from individual time-points (N=3 each) |
| Mukerjee and Ghosh | 1962 | Ann Biochem Exp Med. 22:73-6 | U | U | Vibrio cholerae | **-** | IV | rabbit | U | N | N | N | NO | phage detected in blood, spleen, liver, kidney, lung, bladder, bile, intestinal content and intestinal wall | 1 | 3 | 1 | N unspecified |
| Nakai et al. | 1999 | Dis Aquat Organ. 37(1):33-41 | PLgY-16 | Sipho | Lactococcus | **+** | IP | fish | Y | N | N | N | NO | phage detected in spleen | 1 | 3 | 9 | N represents three different time-points, three fish each |
|  |  |  |  |  |  |  |  |  |  |  |  |  | YES | phage detected in spleen | 1 | 3 | 21 | N represents seven different time-points, three fish each |
|  |  |  |  |  |  |  | oral | fish | Y | 7 | NO | NO | YES | in some fish phage detected in spleen | 1 | 2 | 9 | N represents three different time-points, three fish each |
| Nelstrop  et al. | 1968 | Immunology 14(3):325-37 | T1 | Sipho | Escherichia coli | **-** | IV | rabbit | U | N | N | N | NO | phage detected in blood | 1 | 3 | 6 |  |
|  |  |  | T2 | Myo | Escherichia coli | **-** | IV | rabbit | U | N | N | N | NO | phage detected in blood | 1 | 3 | 6 |  |
| Nelstrop  et al. | 1968 | Immunology 14(3):339-46 | T1 | Sipho | Escherichia coli | **-** | IV | Fish  (goldfish) | U | N | N | N | NO | phage detected in blood | 1 | 3 | 7 | N summarized |
|  |  |  |  |  |  |  |  | Fish  (dogfish) | U | N | N | N | NO | phage detected in blood | 1 | 3 | 5 | N summarized |
|  |  |  |  |  |  |  |  | lamprey | U | N | N | N | NO | phage detected in blood | 1 | 3 | 3 | N summarized |
| Nishikawa  et al. | 2008 | Arch Virol. 153(3):507-15 | KEP10 | Myo | Escherichia coli | **-** | IP | mouse | A | N | N | N | NO | phage detected in blood, spleen, liver, kidney, lung, muscle, bone marrow and brain | 1 | 3 | 3 |  |
|  |  |  | T4 | Myo | Escherichia coli | **-** | IP | mouse | A | N | N | N | NO | phage detected in blood, spleen, liver, kidney, lung, muscle, bone marrow and brain | 1 | 3 | 3 |  |
| Ochs et al. | 1971 | J Clin Invest. 50(12):2559-68 | phiX174 | Micro | Escherichia coli | **-** | IV | human | A | N | N | N | NO | phage detected in blood | 1 | 3 | 5 |  |
| Oechslin et al. | 2017 | J Infect Dis. 215(5):703-712 | Mix: PP1131 Phagoburn | mix | Pseudomonas aeruginosa | **-** | IV | rat | A | N | N | N | NO | phage detected in blood | 1 | 3 | 8 |  |
| Oliveira et al. | 2009 | Poult Sci 88(4):728-33 | phiF258E | Sipho | Escherichia coli | **-** | IM | chicken | Y | N | N | N | NO | phage detected in spleen, and randomly in liver, duodenum and respiratory tract | 1 | 3 | 9 | N summarized from all groups/doses |
|  |  |  |  |  |  |  | oral | chicken | Y | 8 | NO | YES | NO | no phage detected in spleen or liver | 0 | 3 | 3 | random presence of phage in respiratory tract while not detected in spleen or liver, thus possible contamination of respiratory tract |
|  |  |  |  |  |  |  |  |  |  | 7 | NO | YES | NO | no phage detected in spleen or liver | 0 | 3 | 3 | random presence of phage in respiratory tract while not detected in spleen or liver, thus possible contamination of respiratory tract |
|  |  |  |  |  |  |  |  |  |  | 6 | NO | YES | NO | no phage detected in spleen or liver | 0 | 3 | 3 |  |
|  |  |  | phiF61E | Myo | Escherichia coli | **-** | IM | chicken | Y | N | N | N | NO | phage detected in spleen, and randomly in liver, duodenum and respiratory tract | 1 | 3 | 9 | N summarized from all groups/doses |
|  |  |  |  |  |  |  | oral | chicken | Y | 8 | NO | YES | NO | no phage detected in spleen or liver | 0 | 3 | 3 |  |
|  |  |  |  |  |  |  |  |  |  | 7 | NO | YES | NO | no phage detected in spleen or liver | 0 | 3 | 3 | random presence of phage in respiratory tract while not detected in spleen or liver, thus possible contamination of respiratory tract |
|  |  |  |  |  |  |  |  |  |  | 6 | NO | YES | NO | no phage detected in spleen or liver | 0 | 3 | 3 |  |
|  |  |  | phiF78E | Myo | Escherichia coli | **-** | IM | chicken | Y | N | N | N | NO | phage detected in spleen, liver and randomly in duodenum and respiratory tract | 1 | 3 | 9 | N summarized from all groups/doses |
|  |  |  |  |  |  |  | oral | chicken | Y | 8 | NO | YES | NO | phage randomly detected in spleen, liver, duodenum and respiratory tract | 1 | 1 | 3 |  |
|  |  |  |  |  |  |  |  |  |  | 7 | NO | YES | NO | phage randomly detected in spleen, liver, duodenum and respiratory tract | 1 | 1 | 3 |  |
|  |  |  |  |  |  |  |  |  |  | 6 | NO | YES | NO | no phage detected in spleen or liver | 0 | 3 | 3 |  |
| Pagava et al. | 2011 | Georgian Med News. (196-197):101-5 | Mix (Pyobacteriophage, Eliava) | U | Staphylococcus, E. coli, Streptococcus, Pseudomonas, Proteus | **mix** | oral | human | Y | U | U | NO | YES | phage detected in blood in majority of individuals | 1 | 2 | 7 | Blood samples (unspecified overlap with urine samples) |
|  |  |  |  |  |  |  |  | human | Y | U | U | NO | YES | phage detected in urine in majority of individuals | 1 | 2 | 55 | Urine samples  (unspecified overlap with blood samples) |
| Park et al. | 2000 | Appl Environ Microbiol. 66:1416-22 | mix (PPpW-3 and PPpW-4) | mix | Pseudomonas plecoglossicida | **-** | oral | fish | A | 5 | NO | NO | NO | phage detected in kidneys | 1 | 3 | 3 |  |
|  |  |  |  |  |  |  |  |  |  |  |  |  | YES | phage detected in kidneys | 1 | 3 | 3 |  |
| Pouillot et al. | 2012 | Antimicrob Agents Chemother 56(7):3568-75 | EC200PP | Podo | Escherichia coli | **-** | IP | rat | Y | N | N | N | NO | phage detected in blood, spleen, kidney, brain, and urine | 1 | 3 | 10 |  |
|  |  |  |  |  |  |  | SC | rat | Y | N | N | N | NO | phage detected in blood, spleen, kidney, | 1 | 3 | 10 |  |
| Prasad et al. | 2011 | J Environ Biol. 32(2):161-8 | FCP1 | Sipho | Flavobacterium columnare | **-** | IM | fish | Y | N | N | N | YES | phage detected in serum, liver and kidney | 1 | 3 | 10 | phage taxonomy corrected here to Siphoviridae, since designated by Prasad et al. as "Podoviridae" but described as "hexagonal head and non contractile long tail" |
|  |  |  |  |  |  |  | oral | fish | Y | 7 | NO | NO | YES | phage detected in serum, liver and kidney | 1 | 3 | 10 |  |
| Reynaud  et al. | 1992 | Vet Microbiol 30(2-3):203-12 | CF0103 | Podo | Escherichia coli | **-** | oral | rabbit | A | 9 | NO | YES | NO | phage detected in spleen, liver and kidney | 1 | 2 | 2 |  |
| Ryan et al. | 2012 | Eur J Pharm Sci 47(2):297-304 | T4 | Myo | Escherichia coli | **-** | t by microneedles | rat | A | N | N | N | NO | phage detected in spleen and liver | 1 | 3 | 4 |  |
| Samoylova and Smith | 1999 | Muscle Nerve. 22(4):460-6 | M13 | Ino | Escherichia coli | **-** | IV | mouse | A | N | N | N | NO | phage detected in liver, kidney, heart, muscle and brain | 1 | 3 | 1 | phage display study, where a wild type phage was used as a control |
| Schultz and Frohlich | 1965 | Proc Soc Exp Biol Med. 118:136-8 | T5 | Sipho | Escherichia coli | **-** | IV | dog | A | N | N | N | NO | phage detected in blood, urine, and cerebrospinal fluid | 1 | 3 | 16 | for urine penetration n=12, 9/12 positive; for cerebrospinal fluid penetration n=11, 8/11 positive |
| Schultz and Neva | 1965 | J Immunol 94:833-41 | T2 | Myo | Escherichia coli | **-** | IV | mouse | Y | N | N | N | NO | phage detected in blood and occasionally in urine | 1 | 3 | 3 |  |
|  |  |  |  |  |  |  |  | rat | Y | N | N | N | NO | phage detected in blood and occasionally in urine | 1 | 3 | 1 |  |
| Semler et al. | 2014 | Antimicrob Agents Chemother. 58(7):4005-13 | KS12 | Myo | Burkholderia | **-** | IP | mouse | A | N | N | N | NO | phage detected in lungs | 1 | 3 | 10 | mice immunocompromised by cyclophosphamide, N represents two different time-points, five mice each |
| Sigel et al. | 1968 | Proc Soc Exp Biol Med. 128(4):977-9 | T2 | Myo | Escherichia coli | **-** | IM | fish | Y | N | N | N | NO | phage detected in serum | 1 | 3 | 6 | N summarized from two experiments |
|  |  |  |  |  |  |  | IV | fish | Y | N | N | N | NO | phage detected in serum | 1 | 3 | 4 |  |
| Singla et al. | 2015 | J Infect Dis. 212(2):325-34 | KPO1K2 | Podo | Klebsiella pneumoniae | **-** | IP | mouse | A | N | N | N | YES | phage detected in lungs | 1 | 3 | 36 | N represents three different schedules, 12 mice each |
|  |  |  |  |  |  |  |  | mouse | A | N | N | N | YES | phage detected in lungs | 1 | 3 | 36 | N represents three different schedules, 12 mice each, duplication of the experiment |
| Smith and Huggins | 1983 | J Gen Microbiol. 129(8):2659-75 | B44/1 | U | Escherichia coli | **-** | oral | cow | Y | 11 | NO | YES | YES | in some animals phage detected in spleen and blood in low concentration | 1 | 1 | 7 | table 3 of the original text, colostrum deprived calves |
|  |  |  |  |  |  |  |  |  |  |  |  | U | YES | phage infrequently detected in blood | 1 | 1 | 5 | table 7 of the original text, phage applied together with B44/3 |
|  |  |  | B44/2 | U | Escherichia coli | **-** | oral | cow | Y | 11 | NO | YES | YES | phage detected infrequently in spleen and blood in low concentration | 1 | 1 | 7 | table 3 of the original text, colostrum deprived calves |
|  |  |  | B44/3 | U | Escherichia coli | **-** | oral | cow | Y | 11 | NO | U | YES | phage infrequently detected in blood | 1 | 1 | 5 | table 7 of the original text, phage applied together with B44/1 |
| Smith and Huggins | 1982 | J Gen Microbiol 128(2):307-18 | R | U | Escherichia coli | **-** | IM | mouse | A | N | N | N | NO | phage detected in blood, spleen, liver, muscle and brain | 1 | 3 | 15 | N summarized at time points when all organs contained phage |
|  |  |  |  |  |  |  |  | mouse | A | N | N | N | YES | phage detected in blood, spleen and muscle | 1 | 3 | 24 | N summarized at time points when all organs contained phage (phage and bacteria administered in the same time) |
|  |  |  |  |  |  |  |  | mouse | A | N | N | N | YES | phage detected in blood, spleen and muscle | 1 | 3 | 30 | N summarized at time points when all organs contained phage (phage administered 8h later than bacteria) |
|  |  |  |  |  |  |  |  | mouse | A | N | N | N | YES | phage detected in blood, spleen and brain | 1 | 3 | 21 | N summarized at time points when all organs contained phage; bacteria administered intracerebrally |
| Sulkin at al. | 1957 | Science 125(3251):742-3 | 53 | U | Staphylococcus aureus | **+** | IV | rabbit | A | N | N | N | NO | phage detected in blood | 1 | 3 | 6 |  |
| Takemura-Uchiyama  et al. | 2014 | Microbes Infect. 16(6):512-7 | S13` | Podo | Staphylococcus aureus | **+** | IP | mouse | A | N | N | N | NO | phage detected in blood, spleen, liver and lungs | 1 | 3 | 6 |  |
|  |  |  |  |  |  |  |  |  |  |  |  |  | YES | phage detected in blood, spleen, liver and lungs | 1 | 3 | 6 |  |
| Taylor et al. | 1997 | J Immunol 158(2):842-50 | phiX174 | Micro | Escherichia coli | **-** | IV | monkey | A | N | N | N | NO | phage detected in blood | 1 | 3 | 5 |  |
| Tiwari et al. | 2011 | J Microbiol. 49(6):994-9 | PA1phi | Sipho | Pseudomonas aeruginosa | **-** | IP | mouse | A | N | N | N | NO | phage detected in blood, spleen, liver and lungs | 1 | 3 | 12 |  |
|  |  |  |  |  |  |  |  |  |  |  |  |  | YES | phage detected in blood, spleen, liver and lungs | 1 | 3 | 12 |  |
| Trigo et al. | 2013 | PLoS Negl Trop Dis. 7(4):e2183 | D29 | Sipho | Mycobacterium | **N** | IM | mouse | A | N | N | N | YES | phage detected in serum, spleen and draining lymph nodes | 1 | 3 | 5 |  |
| Uchiyama  et al. | 2009 | Microbiol Immunol 53(5):301-4 | KEP10 | Myo | Escherichia coli | **-** | IP | mouse | A | N | N | N | NO | phage detected in blood | 1 | 3 | 9 |  |
|  |  |  | KPP10 | Myo | Escherichia coli | **-** | IP | mouse | A | N | N | N | NO | phage detected in blood | 1 | 3 | 9 |  |
|  |  |  | phiEF24C | Myo | Escherichia coli | **-** | IP | mouse | A | N | N | N | NO | phage detected in blood | 1 | 3 | 9 |  |
|  |  |  | phiMR11 | Sipho | Escherichia coli | **-** | IP | mouse | A | N | N | N | NO | phage detected in blood | 1 | 3 | 9 |  |
| Uhr and Weissmann | 1965 | J Immunol. 94:544-50 | phiX174 | Micro | Escherichia coli | **-** | IV | guinea pig | A | N | N | N | NO | phage detected in liver | 1 | 3 | 1 | N unspecified |
|  |  |  | T2 | Myo | Escherichia coli | **-** | IV | rabbit | A | N | N | N | NO | phage detected in spleen | 1 | 3 | 1 |  |
| Vitiello et al. | 2005 | Virus Res. 2005 Dec;114(1-2):101-3 | lambda | Sipho | Escherichia coli | **-** | IP | mouse | A | N | N | N | NO | phage detected in blood | 1 | 3 | 5 |  |
| Wang et al. | 2016 | Front Microbiol. 7:934 | SLPW | Podo | Staphylococcus aureus | **+** | IP | mouse | A | N | N | N | NO | phage detected in blood, spleen and lung | 1 | 3 | 6 |  |
|  |  |  |  |  |  |  |  |  |  |  |  |  | YES | phage detected in blood, spleen and lung | 1 | 3 | 6 |  |
| Weber-Dąbrowska et al. | 1987 | Arch Immunol Ther Exp (Warsz) 35(5):563-8 | phi131 or 676 | Myo | Staphylococcus aureus | **+** | oral | human | A | 10 | YES | NO | YES | phage detected in blood | 1 | 2 | 20 |  |
|  |  |  | Ps/68 | Myo | Pseudomonas aeruginosa | **-** | oral | human | A | 8 | YES | NO | YES | phage detected in blood | 1 | 2 | 7 |  |
| Wolochow et al. | 1966 | J Infect Dis. 116(4):523-8 | T1 | Sipho | Escherichia coli | **-** | oral | rat | A | 10 | NO | YES | NO | phage detected in intestinal lymph | 1 | 3 | 8 | surgical administration into duodenum, phage administered in mixture with non-sensitive bacteria |
|  |  |  |  |  |  |  |  |  |  | 6 | NO | YES | NO | phage detected in intestinal lymph | 1 | 1 | 8 |  |

**Additional information**

**Table S3**. Stomach pH in species engaged in phage research (modified from Kararli 1995, Marieb and Hoehn 2010, Beasley et al. 2015)

| human | 1.5 (fed: 5.0) |
| --- | --- |
| mouse | 3.8 |
| rabbit | 1.9 |
| rat | 4.4 |
| cow | 4.2 |
| chicken | 3.7 |
| horse | 4.7 |
| sheep | 4.7 |
| dog | 4.5 |
| guinea pig | 4.3 |

**Table S4**. Circulation factors of selected bacteriophages after IV injections: relation of phage titer achieved in blood to expected phage titer; ‘Expected phage titer (A)’ represents hypothetical dilution of phage in animal bodies calculated from body weight; *italics* denote values estimated from graphics of original reports. Values were extracted, or calculated or averaged by the author of this review from original data in referenced reports. *N* – not specified.

| **animal** | **phage** | **phage dose [pfu/animal]** | **expected phage titer    (A)** | **observed phage titer 1-5 min after injection (B)** | **observed phage titer 20-30 min after injection  (C)** | **circulation factor  5 min**  **(B/A)** | **circulation factor  30 min**  **(C/A)** | **reference** |
| --- | --- | --- | --- | --- | --- | --- | --- | --- |
| monkey | phiX174 | 10^11^ | 2x10^7^ | *10^8^* | *10^8^* | **5.000** | **5.000** | Taylor  et al. 1997 |
| rabbit | T1 | 4x10^9^ | 10^6^ | *2.5x10^7^ (averaged)* | *6x10^4^ (averaged)* | **25.000** | **0.060** | Nelstrop  et a. 1968,  Fig 1 and 2 |
| rabbit | *N* | 10^9^ | 2.5x10^5^ | *10^5^* | *1.2x10^4^* | **0.400** | **0.048** | Sulkin  et al. 1957 |
| mice | T2 | 2.5x10^11^ (averaged) | 2.5x10^10^ | *1.96x10^9^* | *1.6x10^8^* | **0.078** | **0.006** | Schultz and Neva 1965 |
| mice | T4 | 5x10^8^ | 2.5x10^7^ | *10^7^* | *10^6^* | **0.400** | **0.040** | Inchley 1969 |
| rat | T2 | 10^11^ | *4.4x10^8^* | *2x10^8^* | *10^8^* | **0.454** | **0.227** | Schultz and Neva 1965 |
| dog | T5 | 5x10^10^ (averaged) | 3.3x10^6^ | ^-^ | 2.7x10^5^ | **-** | **0.082** | Schultz and Frochlich 1965 |
| dog | *N* | 10^11^ | 8x10^6^ | 3x10^6^ | 6x10^5^ | **0.375** | **0.075** | Keller and Zatzman 1959 |
| **median circulation factor (min-max)** | | | | | | **0.400  (0.078-25)** | **0.068  (0.006-5)** |  |

**Table S5**. Average body weights of vertebrates used for phage doses normalization (N/A – not applicable for this review, * – according to an analyzed report)

| **Animals** | **Average body weight [kg]** | |
| --- | --- | --- |
|  | **young** | **adult** |
| Chicken | 0.2 | 1.5 |
| Cow | 70 | N/A |
| Fish | 10 or 20* | N/A |
| Human | 30 | 80 |
| Mouse | N/A | 0.02 |
| Pig | 10 | N/A |
| Quail | 0.1 | N/A |
| Rabbit | N/A | 4 |
| Rat | N/A | 0.3 |
| Sheep | 10 | 100 |

**Figure S1**. Bacteriophage clearance from blood in rodent models: fraction of initial phage titer in time. Data for calculation were extracted only from reports where IV injection of phage was applied to exclude potential effect of phage retention in site of injection. From each report 1 or 2 experiments were extracted (Inchley 1969, Kim et al. 2008, Oechslin et al. 2016). Median values and a trend line are presented in a semi-log plot, the equation of the trend line was used to assess half-life of phage (see main text, Phage clearance section).


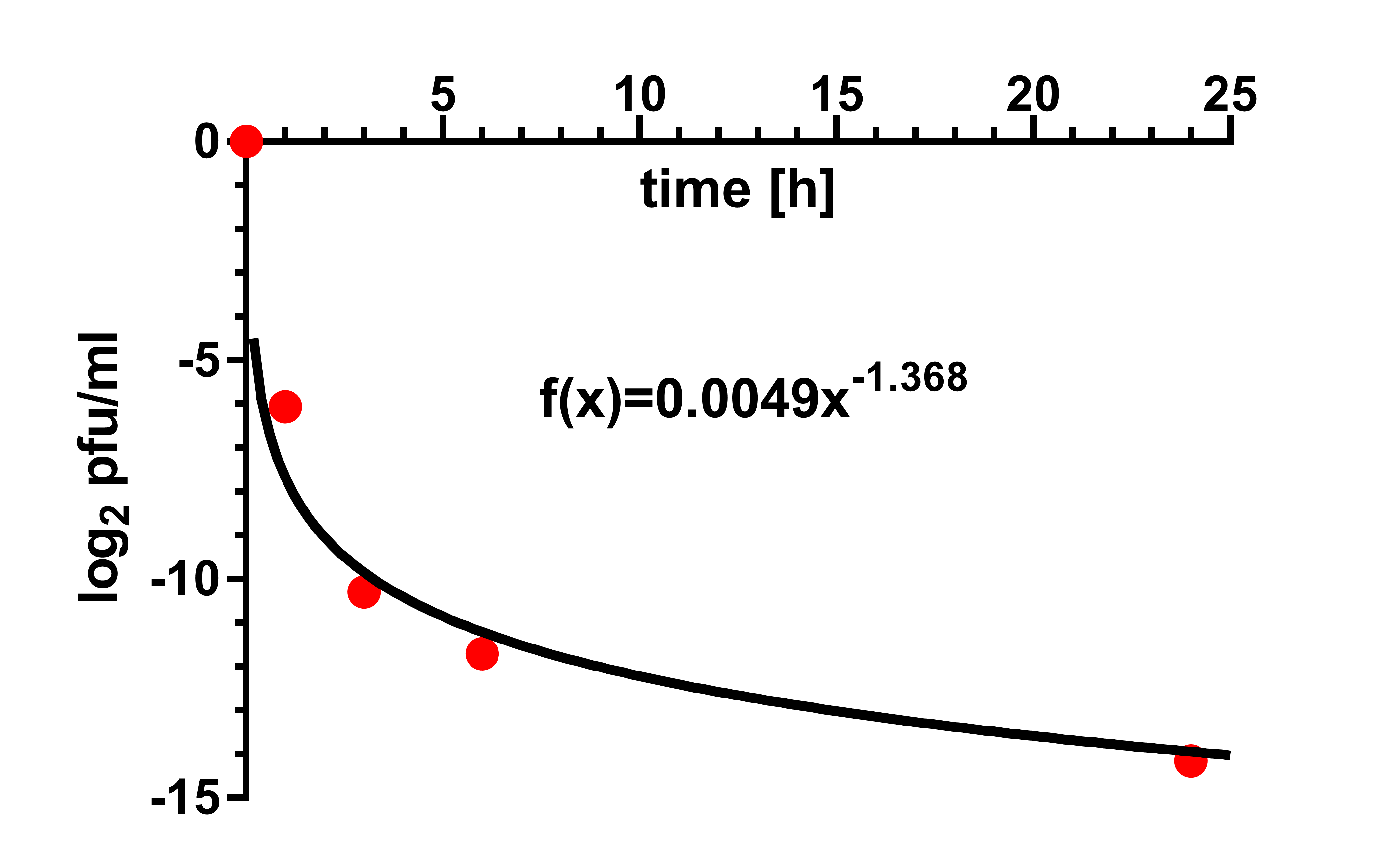


**References**

Beasley DE, Koltz AM, Lambert JE, Fierer N, Dunn RR. 2015. The Evolution of Stomach Acidity and Its Relevance to the Human Microbiome. PLoS One 29:e0134116.

Inchley CJ. 1969. The actvity of mouse Kupffer cells following intravenous injection of T4 bacteriophage. Clin Exp Immunol 5:173-87.

Kararli TT. 1995. Comparison of the gastrointestinal anatomy, physiology, and biochemistry of humans and commonly used laboratory animals. Biopharm Drug Dispos 16:351-80.

Keller R, Zatzman ML. 1959. Studies on the Factors Concerned in the Disappearance of Bacteriophage Particles from the Animal Body. The Journal of Immunology 83: 167-172.

Kim KP, Cha JD, Jang EH, Klumpp J, Hagens S, Hardt WD, Lee KY, Loessner MJ. 2008. PEGylation of bacteriophages increases blood circulation time and reduces T-helper type 1 immune response. Microb Biotechnol 1:247-57.

Marieb EN, Hoehn K. 2010. Human Anatomy & Physiology. Benjamin Cummings, San Francisco, USA.

Nelstrop AE, Taylor G, Collard P. 1968. Studies on phagocytosis. I. Antigen clearance studies in rabbits. Immunology 14:325-37.

Oechslin F, Piccardi P, Mancini S, Gabard J, Moreillon P, Entenza JM, Resch G, Que YA. 2017. Synergistic interaction between phage therapy and antibiotics clears Pseudomonas aeruginosa infection in endocarditis and reduces virulence. J Infect Dis 215:703-712

Schultz I, Frohlich E. 1965. Viruria and viraliquoria in the dog after intravenous injection of t5 bacteriophage. Proc Soc Exp Biol Med 118:136-8.

Schultz I, Neva FA. 1965. Relationship between blood clearance and viruria after intravenous injection of mice and rats with bacteriophage and polioviruses. J Immunol 94:833-41.

Sulkin SE, Finkelstein RA, Rosenblum ED. 1957. Effect of zymosan on bacteriophage clearance. Science 125:742-3.

Taylor RP, Martin EN, Reinagel ML, Nardin A, Craig M, Choice Q, Schlimgen R, Greenbaum S, Incardona NL, Ochs HD. 1997. Bispecific monoclonal antibody complexes facilitate erythrocyte binding and liver clearance of a prototype particulate pathogen in a monkey model. J Immunol 159:4035-44.
